# Stability and function of adult vasculature is sustained by Akt/Jagged1 signalling axis in endothelium

**DOI:** 10.1038/ncomms10960

**Published:** 2016-03-14

**Authors:** Bethany A. Kerr, Xiaoxia Z. West, Young-Woong Kim, Yongzhong Zhao, Miroslava Tischenko, Rebecca M. Cull, Timothy W. Phares, Xiao-Ding Peng, Jeremiah Bernier-Latmani, Tatiana V. Petrova, Ralf H. Adams, Nissim Hay, Sathyamangla V. Naga Prasad, Tatiana V. Byzova

**Affiliations:** 1 Department of Molecular Cardiology, Joseph J. Jacobs Center for Thrombosis and Vascular Biology, Lerner Research Institute, Cleveland Clinic, Cleveland, Ohio 44195, USA; 2 Department of Neurosciences, Lerner Research Institute, Cleveland Clinic, Cleveland, Ohio 44195, USA; 3 Department of Biochemistry and Molecular Genetics, University of Illinois College of Medicine at Chicago, Chicago, Illinois 60607, USA; 4 Department of Fundamental Oncology, Centre Hospitalier Universitaire Vaudois (CHUV) and University of Lausanne, Lausanne CH-1066, Switzerland; 5 Department of Tissue Morphogenesis, Max Planck Institute for Molecular Biomedicine, Münster 48149, Germany; 6 Faculty of Medicine, University of Münster, Münster 48149, Germany

## Abstract

The signalling pathways operational in quiescent, post-development vasculature remain enigmatic. Here we show that unlike neovascularization, endothelial Akt signalling in established vasculature is crucial not for endothelial cell (EC) survival, but for sustained interactions with pericytes and vascular smooth muscle cells (VSMCs) regulating vascular stability and function. Inducible endothelial-specific Akt1 deletion in adult global Akt2KO mice triggers progressive VSMC apoptosis. In hearts, this causes a loss of arteries and arterioles and, despite a high capillary density, diminished vascular patency and severe cardiac dysfunction. Similarly, endothelial Akt deletion induces retinal VSMC loss and basement membrane deterioration resulting in vascular regression and retinal atrophy. Mechanistically, the Akt/mTOR axis controls endothelial Jagged1 expression and, thereby, Notch signalling regulating VSMC maintenance. Jagged1 peptide treatment of Akt1ΔEC;Akt2KO mice and Jagged1 re-expression in Akt-deficient endothelium restores VSMC coverage. Thus, sustained endothelial Akt1/2 signalling is critical in maintaining vascular stability and homeostasis, thereby preserving tissue and organ function.

## Abstract (web summary)

The Akt pathway integrates multiple signals, but whether it affects vasculature function is debatable. Here the authors show that Akt pathway shutdown in adult mouse endothelium causes destabilization of vasculature leading to cardiac and retinal dysfunction, due to decreased levels of Jagged1 and impaired Notch signaling.

## Graphical abstract

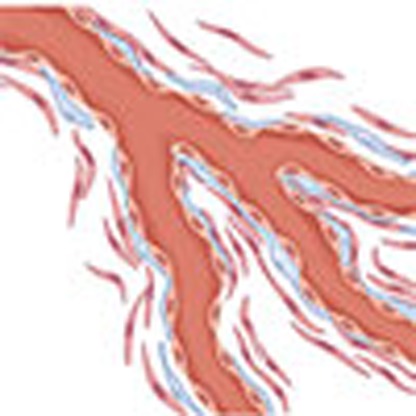

Despite the progress in understanding developmental mechanisms, the signalling pathways operational in postnatal life remain elusive. Gene knockouts (KOs) controlling basic cellular responses result in early lethality or functional compensation by other related molecules, thereby precluding or obscuring detailed analysis of their consequences^1^,^2^,^3^,^4^. While some signalling circuits are shared by developmental and postnatal processes^5^, recent studies using new inducible and/or tissue-specific KO models revealed that the functions of many key signalling molecules in development are distinct and even opposite from that in postnatal life^6^,^7^. In particular, this applies to vasculature, which, once formed during embryonic development, remains relatively quiescent throughout adulthood. With rare exceptions, postnatal expansion or disturbance of vasculature is triggered by injury or pathological conditions^8^. Therefore, the pathways mediating dynamic cellular responses in development, such as vascular cell differentiation, migration and proliferation, are expected to be dormant during adulthood. An essential part of vasculature is endothelium, a crucial interface between circulating blood and tissues, and interference with its survival or integrity leads to pathologies ranging from acute thrombosis to uncontrolled inflammation^8^. Thus, it is critical for healthy endothelium to remain quiescent and unperturbed.

One main pathway activated during growth, cell proliferation and migration is Akt signalling, which integrates growth factor, cytokine and metabolic stimuli, and is essential for development^9^,^10^. Akt mutations in *Drosophila* or substantial reductions in Akt activity, such as those observed in double KO of Akt1 and Akt2 in mice, result in lethality^11^,^12^. In endothelium, Akt signalling is activated by growth factor stimulation during angiogenesis^9^,^13^. While no vascular defects were observed in ubiquitous single Akt2KO^9^, Akt1KO mice displayed increased endothelial apoptosis under pathological conditions^14^ and a series of abnormalities during vascular development and angiogenesis, some of which were highly context dependent^13^,^14^,^15^,^16^,^17^,^18^,^19^. Overall, the exact functions of Akt signalling in vasculature remain under debate^13^, mostly due to the difficulties in interpreting global AktKO phenotypes, which affected diverse functions of multiple cell types, ranging from endothelial and mural cells to inflammatory cells^14^,^20^, platelets^17^ and fibroblasts^18^,^21^,^22^, involved in neovascularization. Since Akt is positioned at the crossroads of numerous signalling pathways with a number of positive and negative feedback loops^9^,^23^,^24^,^25^,^26^, thus controlling diverse and even opposite functions, it is impossible to predict the tissue-specific responses to Akt ablation^27^,^28^. A similar scenario applies to one of the upstream activators of Akt, PI3K signalling, and its role in vasculature. While most studies conclude that this signalling axis is absolutely critical for vascular development^29^,^30^,^31^, interference with PI3K signalling either diminishes^30^ or promotes neovascularization^32^,^33^,^34^.

Since the Akt pathway is often hyperactivated in proliferating and metastatic cancer cells^10^, several potent Akt inhibitors are currently in clinical trials in patients with advanced malignancies^35^,^36^,^37^. However, the consequences of Akt signalling ablation and the nature of observed adverse effects in humans remain poorly understood^35^,^37^,^38^. The question whether the Akt pathway, typically associated with cell transformation and proliferation, is essential for normal cells existing in a relatively quiescent state, exemplified by endothelium, remains open.

Accordingly, to determine whether Akt signalling remains important for established vasculature and to understand the Akt-dependent circuits operational in adults, we induced Akt1 deletion in endothelial cells (ECs) of mature animals (Akt1ΔEC). To exclude possible compensation by Akt2, which might affect total Akt activity^39^, we also generated double Akt1ΔEC;Akt2KO mice. On the basis of the results of developmental studies, one might predict that the shutdown of Akt activity in any cell type is likely to lead to excessive apoptosis. It could be anticipated that disruption of Akt signalling in EC might result in the loss of endothelial integrity, subsequent thrombosis and sudden death, similar to the phenotype observed by the disruption of the best known Akt activator in endothelium, vascular endothelial growth factor (VEGF)^40^. We show that EC Akt deletion does not alter EC survival, but instead leads to the gradual loss of VSMCs due to diminished Jagged1/Notch signalling. These structural changes in the vasculature result in impaired vessel perfusion and severe tissue dysfunction. Thus, Akt signalling is integral in maintaining tissue function by preserving postnatal vessel integrity.

## Results

### Endothelial Akt1 deletion triggers vascular regression

Akt1 excision was induced in mature Akt1ΔEC;Akt2KO and Akt1ΔEC mice with wild-type (WT) and Akt2KO mice serving as controls. As detailed in Methods and in Supplementary Figs 1–3, deletion was most complete in hearts and retinas resulting in more than 95% ablation of Akt1 and nearly complete deletion of total Akt activity in endothelium of Akt1ΔEC;Akt2KO mice.

**Figure 1:**
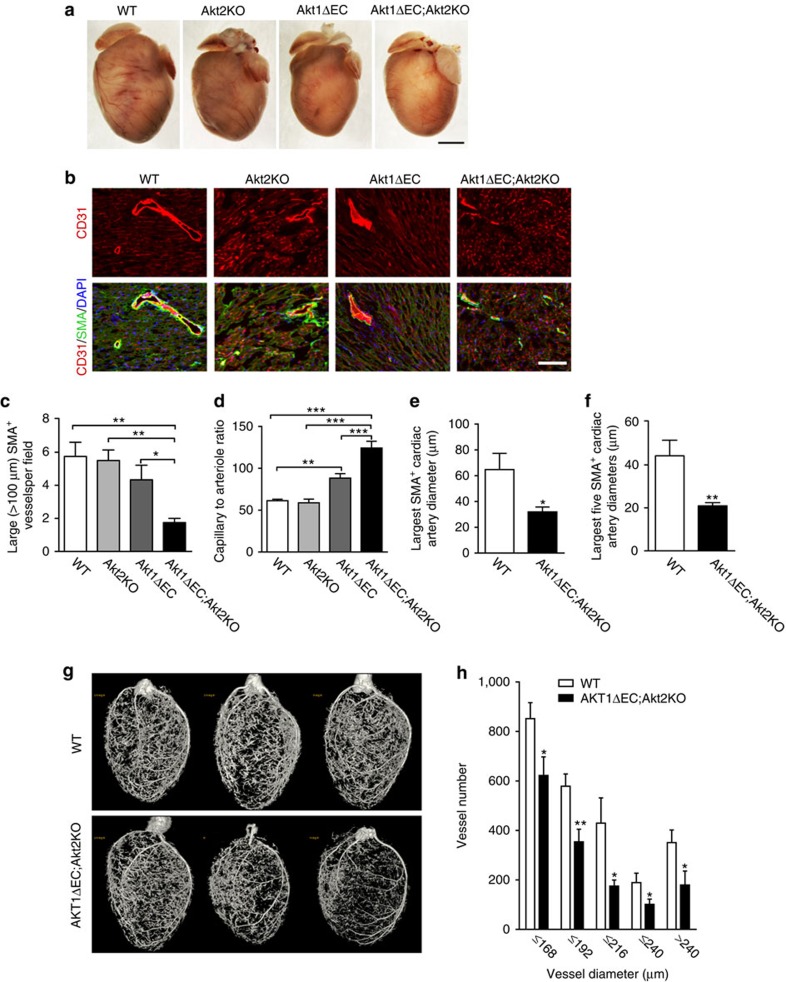
Vascular abnormalities triggered by Akt1 deletion. (**a**) Representative images of WT, Akt2KO, Akt1ΔEC and Akt1ΔEC;Akt2KO hearts after tamoxifen treatment for 4 weeks (5 days i.p. tamoxifen injection followed by 3 weeks tamoxifen diet, 8–9 weeks old). Scale bar, 2 mm. (**b**) Representative images of WT, Akt2KO, Akt1ΔEC and Akt1ΔEC;Akt2KO heart sections after 4 weeks of tamoxifen treatment stained for CD31 (red), SMA (green) and DAPI (blue). Scale bar, 100 μm. (**c**,**d**) Quantification of >100 μm (large) SMA^+^ vessels per field and capillary (CD31^+^ SMA^−^) to arteriole (CD31^+^ SMA^+^) ratio represented as mean±s.e.m. (*n*=4). (**e**,**f**) Quantification of the largest and top five largest cardiac artery diameters measured in the heart sections represented as mean±s.e.m. (*n*=5–6). (**g**) Representative microCT images for WT and Akt1ΔEC;Akt2KO hearts after 10 weeks of tamoxifen treatment (5 days of i.p. tamoxifen injection followed by 9 weeks of tamoxifen diet, 14–15 weeks old). (**h**) Quantification of the numbers of vessels within each diameter bin represented as mean±s.e.m. (*n*=5–7). *P<0.05, **P<0.01 and ***P<0.005 by one-way analysis of variance (**c**,**d**) or Student's *t*-test (**e**–**h**).

Surprisingly, Akt shutdown in adult endothelium did not cause acute events leading to lethality. However, gross examination of Akt1ΔEC;Akt2KO and Akt1ΔEC hearts revealed visibly fewer and smaller coronary vessels compared with WT and Akt2KO hearts (Fig. 1a). To further assess the changes in vasculature, we stained heart sections with CD31 and smooth muscle actin (SMA) to visualize ECs and mural cells, respectively (Fig. 1b). After 4 weeks of excision, there was a >3-fold decrease in the density of large (>100 μm) SMA^+^ blood vessels in Akt1ΔEC;Akt2KO hearts compared with both WT and Akt2KO hearts (Fig. 1c). In Akt1ΔEC mice, large SMA^+^ blood vessel numbers were also slightly decreased compared with WT, indicating the contribution of both Akt isoforms (Fig. 1b,c). Surprisingly, there was no loss of ECs, and the density of small CD31^+^ capillaries even increased in Akt1ΔEC;Akt2KO hearts (Fig. 1b) resulting in >2-fold higher capillary to arteriole ratios in Akt1ΔEC;Akt2KO hearts than in WT and Akt2KO hearts (Fig. 1d). Concurrently, no marked changes were observed in vasculature of control WT or Akt2KO mice (Fig. 1b–d). Quantitative analysis revealed that the diameters of SMA-positive arterioles in hearts were substantially reduced on Akt excision (Fig. 1e,f).

Microcomputed tomography (microCT) imaging confirmed the structural changes in arterial vasculature of WT and Ak1ΔEC;Akt2KO hearts (Fig. 1g). Analysis of the numbers of vessels in each diameter bin demonstrated the loss of larger arteries and arterioles (Fig. 1h). However, the smaller capillaries, evident by staining, are not perfused by the contrast reagent; therefore, the capillary increase could not be detected by microCT (Supplementary Fig. 4). The use of a vasodilator in microCT experiments confirmed that observed changes in the diameter and large blood vessel numbers reflect vascular remodelling rather than vasoconstriction. Of note, ∼30% of Akt1ΔEC;Akt2KO, but not WT mice, experience vascular ruptures during perfusion highlighting their vascular abnormalities. Together, these results show that endothelial Akt1 ablation does not result in the loss of endothelium, but induces severe vascular remodelling characterized by arteriole loss and increased small capillary density. Though Akt2 might partially compensate for the loss of Akt1, it is endothelial Akt1 that is a determining factor in this process.

In parallel, the size and number of cardiomyocytes were assessed in heart sections. Cardiomyocyte area and perimeter showed a small but significant increase in Akt1ΔEC;Akt2KO hearts compared with WT (Supplementary Fig. 5). However, the number of cardiomyocytes per field was not significantly changed demonstrating that the vascular changes were not due to alterations in heart size.

**Figure 2:**
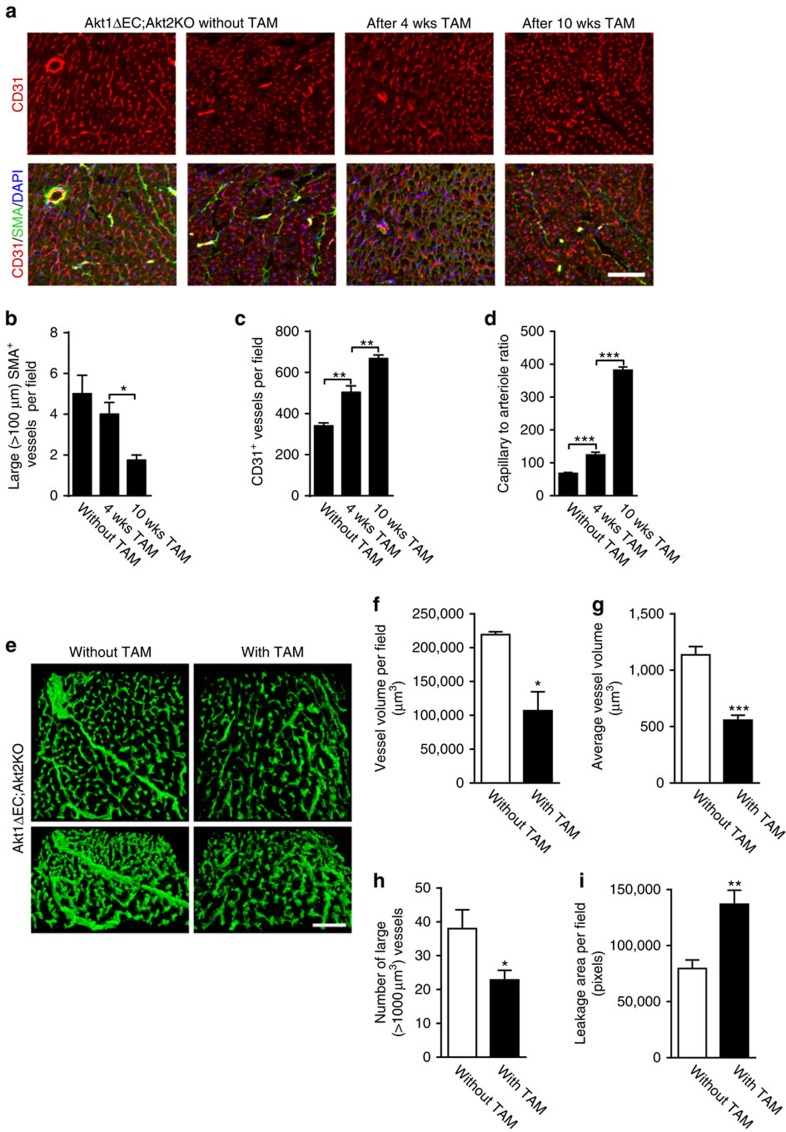
Vascular regression and vascular patency on tamoxifen administration. (**a**) Representative images of Akt1ΔEC;Akt2KO 13- to- 14-week-old mice heart sections stained for CD31 (red), SMA (green) and DAPI (blue) without tamoxifen (TAM), and 4 and 10 weeks (wks) after Akt1 excision (5 days of i.p. tamoxifen injection followed by 3 or 9 weeks of tamoxifen diet). Scale bar, 100 μm. (**b**–**d**) Quantification of >100 μm (large) SMA^+^ or CD31^+^ vessels per field and capillary (CD31^+^ SMA^−^) to arteriole (CD31^+^ SMA^+^) ratio represented as mean±s.e.m. (*n*=4). (**e**) Representative images of Akt1ΔEC;Akt2KO hearts of littermate mice perfused with lectin with and without TAM treatment for 4 weeks. Scale bar, 100 μm. (**f**–**i**) Quantification of vessel volume per field, average vessel volume, large vessel number (>1,000 μm^3^) and leakage area per field represented as mean±s.e.m. (*n*=3–5). *P<0.05, **P<0.01 and ***P<0.005 by one-way analysis of variance (**b**–**d**) or Student's *t*-test (**f**–**i**).

To examine the temporal effects of Akt deficiency, we monitored changes in hearts of Akt1ΔEC;Akt2KO mice without or 4 and 10 weeks after tamoxifen-induced excision. As evident from Fig. 2a,b, there was a time-dependent loss of large SMA^+^ blood vessels, which resulted in 3-fold lower density of large arterioles (>100 μm) in the hearts of Akt1ΔEC;Akt2KO mice but not in WT and Akt2KO mice after 10 weeks of tamoxifen treatment. Density of CD31^+^ capillaries displayed an opposite trend (Fig. 2c) thereby increasing the capillary to large arteriole ratio by 2.5- and 5.5-fold in Akt1ΔEC;Akt2KO hearts, 4 and 10 weeks after initiation of Akt1 deletion, respectively (Fig. 2d). Thus, endothelial Akt1 deletion results in a progressive loss of larger and mature SMA^+^ blood vessels, despite the overall high density of capillaries. In contrast, lymphatic vasculature in Akt1ΔEC;Akt2KO mice was not significantly diminished compared with WT as judged by LYVE1 staining (Supplementary Fig. 6).

### Vascular patency is diminished after Akt1 excision

To further determine how these changes affect vascular patency of microvasculature, we perfused Akt1ΔEC;Akt2KO littermate mice (without and 4 weeks after excision) with lectin, which penetrates capillaries and larger blood vessels. Consistent with microCT, confocal imaging revealed marked changes in functional cardiac vasculature on Akt deletion (Fig. 2e and Supplementary Movies 1 and 2). EC Akt deletion resulted in a 55% decrease of perfused vasculature volume (Fig. 2f) and 60% reduction in the average volume of functional blood vessels (Fig. 2g). As evident from Fig. 2h, there was a marked loss of large blood vessels (>1,000 μm^3^ in volume) on tamoxifen treatment. Thus, despite the high density of small CD31^+^ capillaries, the volume of patent or perfused vasculature in hearts was severely diminished. Increased lectin in the extravascular space (Fig. 2i) indicated possible vascular leakage in Akt1ΔEC;Akt2KO mice, which might be explained by the loss of VSMC. However, no substantial haemorrhaging was observed in retinas or hearts. Also, theblood–brain barrier (BBB) and blood–spinal cord barrier were intact in Akt1ΔEC;Akt2KO mice (Supplementary Fig. 7), and no clinical signs of neurological dysfunction, such as abnormal gait or seizures were observed.

**Figure 3:**
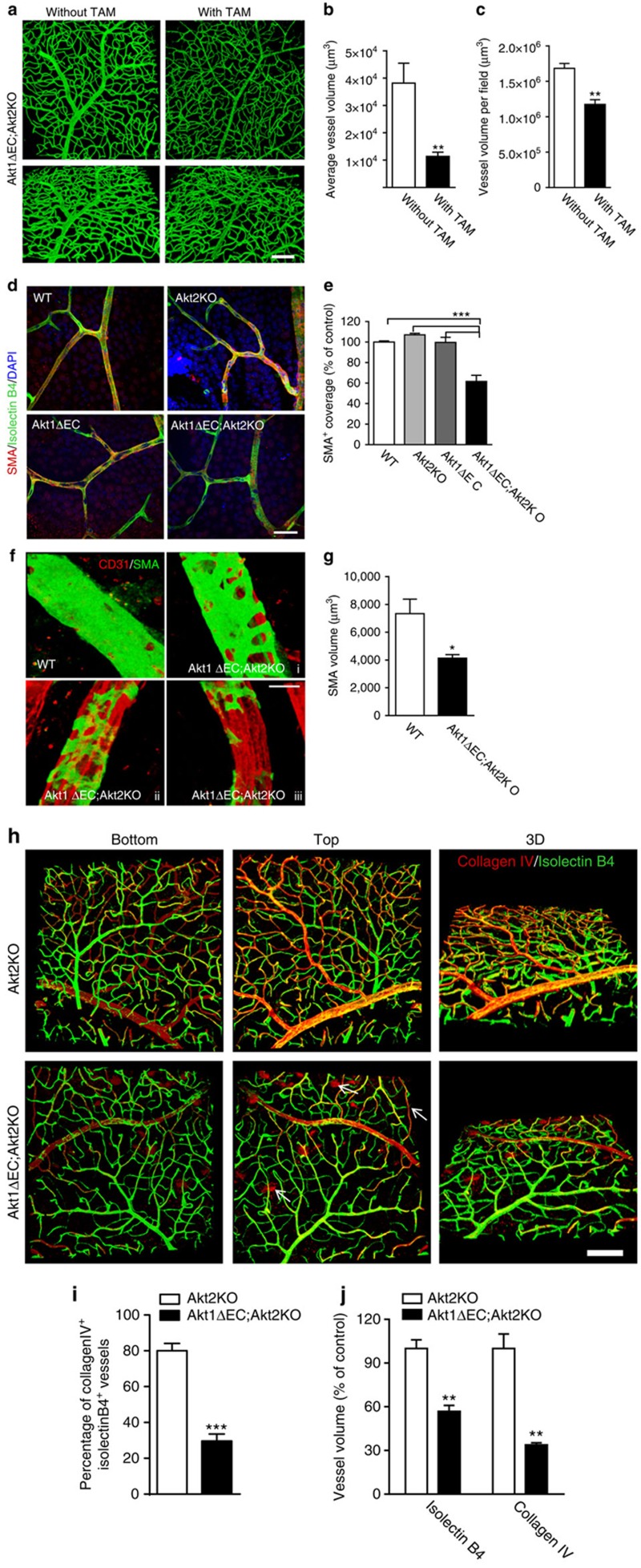
Perfusion and regression of retinas after Akt1 deletion. (**a**) Representative images of Akt1ΔEC;Akt2KO retinas of littermate mice (8–9 weeks of age) perfused with lectin with and without tamoxifen (TAM) treatment for 4 weeks. Scale bar, 100 μm. (**b**,**c**) Quantification of average vessel volume and vessel volume per field represented as mean±s.e.m. (*n*=3). (**d**) Representative images of WT, Akt2KO, Akt1ΔEC and Akt1ΔEC;Akt2KO whole-mount retinas after 4 weeks of tamoxifen treatment (8–9 week old mice) stained for SMA (red), isolectin B4 (green) and DAPI (blue). Scale bar, 50 μm. (**e**) Quantification of per cent SMA^+^ coverage represented as mean±s.e.m. (*n*=3). (**f**) Representative images of WT and three different Akt1ΔEC;Akt2KO whole-mount retinas of 10-day neonatal mice after tamoxifen treatment stained for SMA (green) and CD31 (red) demonstrating the various coverage phenotypes. Scale bar, 200 μm. (**g**) Quantification of the SMA coverage volume per field represented as mean±s.e.m. (*n*=3). (**h**) Representative images of Akt2KO and Akt1ΔEC;Akt2KO whole-mount retinas (8- to 9-week-old mice) after 4 weeks of tamoxifen treatment stained for collagen IV (red) and isolectin B4 (green). The proximal focal plane (top), distal focal plane (bottom) and 3D reconstruction are shown. Arrows demonstrate empty collagen IV sleeves. Scale bar, 100 μm. (**i**,**j**) Quantifications of the percentage of co-patterning, stained vessel volume and thickness of lectin^+^ vessels represented as mean±s.e.m. (*n*=3–4). *P<0.05, **P<0.01 and ***P<0.005 by Student's *t*-test (**b**,**c**,**g**–**j**) or one-way analysis of variance (**e**).

Similar remodelling in patent vasculature was observed in retinas. As shown in Fig. 3a and Supplementary Movies 3 and 4, tamoxifen treatment resulted in a 3.3-fold decrease in the average blood vessel volume (Fig. 3b), which diminished total volume of perfused vasculature (Fig. 3c). These results were corroborated with an analysis of SMA and isolectin B4 co-staining in WT, Akt2KO, Akt1ΔEC and Akt1ΔEC;Akt2KO retinas after tamoxifen treatment (Fig. 3d). Akt1ΔEC;Akt2KO mice exhibited a 50% loss of VSMC coverage compared with WT mice, while changes in Akt2KO and Akt1ΔEC retinas were not significant (Fig. 3e). Diminished VSMC coverage was more apparent when Akt excision was induced at P2 during retinal vasculature development. Figure 3f shows examples of patchy radial artery SMA staining in Akt1ΔEC;Akt2KO retinas at P10, in contrast to complete and smooth VSMC coverage in WT mice. Quantification revealed 50% decrease in VSMC volume in Akt1ΔEC;Akt2KO retinas as compared with WT (Fig. 3g).

Vascular deterioration in Akt1ΔEC;Akt2KO mice was accompanied by a marked loss of the basement membrane component, collagen IV (Fig. 3h and Supplementary Movies 5 and 6). The presence of collagen IV^+^ and isolectin^−^ structures demonstrate areas of vascular regression (Fig. 3h, arrows). Quantification of three-dimensional (3D) images revealed a 3-fold decrease in co-patterning of isolectin and collagen IV in Akt1ΔEC;Akt2KO retinas compared with Akt2KO mice (Fig. 3i). The volume of vasculature with detectable basement membrane was also diminished by 2.6-fold (Fig. 3i). There was a thinning of blood vessels in Akt1ΔEC;Akt2KO retinas compared with Akt2KO (Fig. 3j). Thus, EC-specific Akt excision does not affect endothelium but causes the loss of VSMC coverage, basement membrane and vascular patency in highly vascularized tissues such as hearts and retinas.

### Loss of VSMCs on endothelial Akt excision

**Figure 4:**
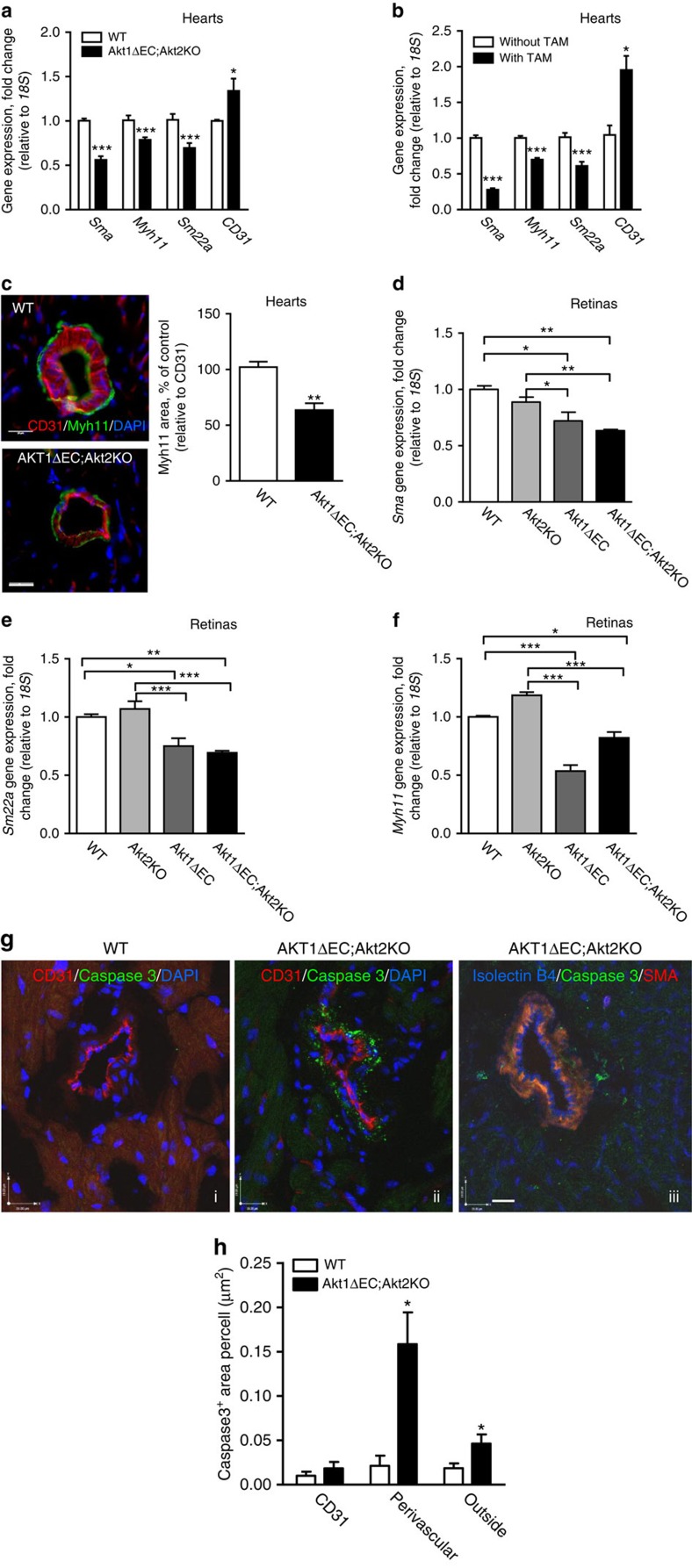
VSMC de-differentiation and apoptosis. (**a**) Gene expression normalized to *18S* in WT and Akt1ΔEC;Akt2KO hearts after 4 weeks of tamoxifen treatment represented as mean fold change from WT±s.e.m. (*n*=4). (**b**) Gene expression normalized to *18S* in Akt1ΔEC;Akt2KO hearts from littermate mice with and without tamoxifen (TAM) treatment for 4 weeks represented as mean fold change from mice without TAM±s.e.m. (*n*=3). (**c**) Representative images of WT and Akt1ΔEC;Akt2KO heart sections after 4 weeks of tamoxifen treatment stained for CD31 (red), Myh11 (green) and DAPI (blue), and quantification of the CD31/Myh11 staining ratio represented as mean±s.e.m. (*n*=3). Scale bar, 25 μm. (**d**) *Myh11* gene expression normalized to *18S* in WT, Akt2KO, Akt1ΔEC and Akt1ΔEC;Akt2KO retinas from mice after 4 weeks of tamoxifen treatment represented as mean fold change from WT±s.e.m. (*n*=6). (**e**,**f**) *Sma* and *Sm22a* gene expression normalized to *18S* in WT, Akt2KO, Akt1ΔEC and Akt1ΔEC;Akt2KO retinas from mice after 4 weeks of tamoxifen treatment represented as mean fold change from WT±s.e.m. (*n*=6). (**g**) Representative images of WT and Akt1ΔEC;Akt2KO heart sections after 10 weeks of tamoxifen treatment stained for CD31 or SMA (red), cleaved caspase 3 (green) and isolectin or DAPI (blue). Scale bar, 19 μm. (**h**) Quantification of the cleaved caspase 3-positive area per cell represented as mean±s.e.m. (*n*=3). *P<0.05, **P<0.01 and ***P<0.005 by Student's *t*-test (**a**–**c**) or one-way analysis of variance (**d**–**f**).

Quantitative reverse transcription–PCR (RT–PCR) analysis of whole-heart lysates confirmed the drastic reduction in VSMC marker expression: *Sma*, *Myh11* and *Sm22a*, in Akt1ΔEC;Akt2KO mice compared with WT, and simultaneous increase in *CD31* gene expression (Fig. 4a). These changes in VSMC differentiation marker expression were triggered by Akt1 excision (Fig. 4b). Heart tissue section staining for Myh11 confirmed the loss of this VSMC differentiation marker in Akt1ΔEC;Akt2KO hearts at the protein level (Fig. 4c). There was almost 50% decrease in the ratio of Myh11^+^ to CD31^+^ area in Akt1ΔEC;Akt2KO hearts compared with WT (Fig. 4c, right panel). Concurrently, 3D reconstruction of NG2-, SMA- and isolectin B4-stained 100-μm heart sections revealed no substantial abnormalities in associations between pericytes and blood vessels in Akt1ΔEC;Akt2KO mice compared with WT (Supplementary Fig. 8A–C). No reduction in pericyte numbers was observed either (Supplementary Fig. 8D–F). Thus, Akt1 KO in EC triggers progressive loss of VSMC differentiation markers while pericyte numbers are not affected.

Similar results were obtained in retinas, where quantitative RT–PCR showed ∼2-fold decline in *Sma* in Akt1ΔEC;Akt2KO mice, while mice with single Akt1ΔEC isoform deletion also had decreased *Sma* (Fig. 4d), thereby confirming the key role for endothelial Akt1. Likewise, Akt1 deletion in endothelium was sufficient to cause reduction in *Sm22a* and *Myh11* expression in retinas (Fig. 4e,f). This phenotype was recapitulated in mice treated with Akt inhibitor, MK2206, which blocks both Akt1 and Akt2 and to a lesser extent, Akt3. Akt inhibition led to decreased expression of VSMC differentiation markers *Sma*, *Sm22a* and *Myh11* in hearts (Supplementary Fig. 9A) and to lesser extent in retinas (Supplementary Fig. 9B). Likewise, there was a reduction in average diameter of retinal blood vessels in mice treated with MK2206 (Supplementary Fig. 9C).

To further understand the mechanisms of VSMC loss in Akt1ΔEC;Akt2KO vasculature, we stained heart tissue sections for cleaved caspase 3. ECs were identified based on CD31 or isolectin B4 staining, whereas VSMC was visualized by SMA staining (Fig. 4g). Notably, most cleaved caspase 3 staining was localized in the area surrounding CD31^+^ ECs (Fig. 4g, ii), which in some cases was also positive for SMA (Fig. 4g, iii). Knowing that Akt deficiency led to VSMC marker loss, first being SMA as early as 4 weeks after initiation of excision, we quantified cleaved caspase 3 in the perivascular area immediately adjacent to CD31^+^ ECs (Fig. 4h). This analysis revealed ∼6-fold increased perivascular cleaved caspase 3 staining in Akt1ΔEC;Akt2KO compared with WT hearts 10 weeks after Akt1 excision (Fig. 4g,h). Simultaneously, cleaved caspase 3 immunoreactivity in the CD31^+^ area was minimal and not significantly different between the two groups (Fig. 4h). Cleaved caspase 3 staining outside of vasculature was moderately increased in Akt1ΔEC;Akt2KO compared with WT hearts, indicating slightly increased apoptosis of other cells, including cardiomyocytes, possibly due to the lack of perfusion (Fig. 4h). Thus, Akt1 deletion in endothelium leads to the loss of VSMC markers followed by apoptosis at the later time points. The process of vascular regression occurred gradually over the period of 10 weeks without marked increases in tissue hypoxia based on pimonidazole staining (Supplementary Fig. 10) consistent with limited cardiomyocyte apoptosis at chosen time points.

### Akt1 signalling controls the Jagged1/Notch pathway

**Figure 5:**
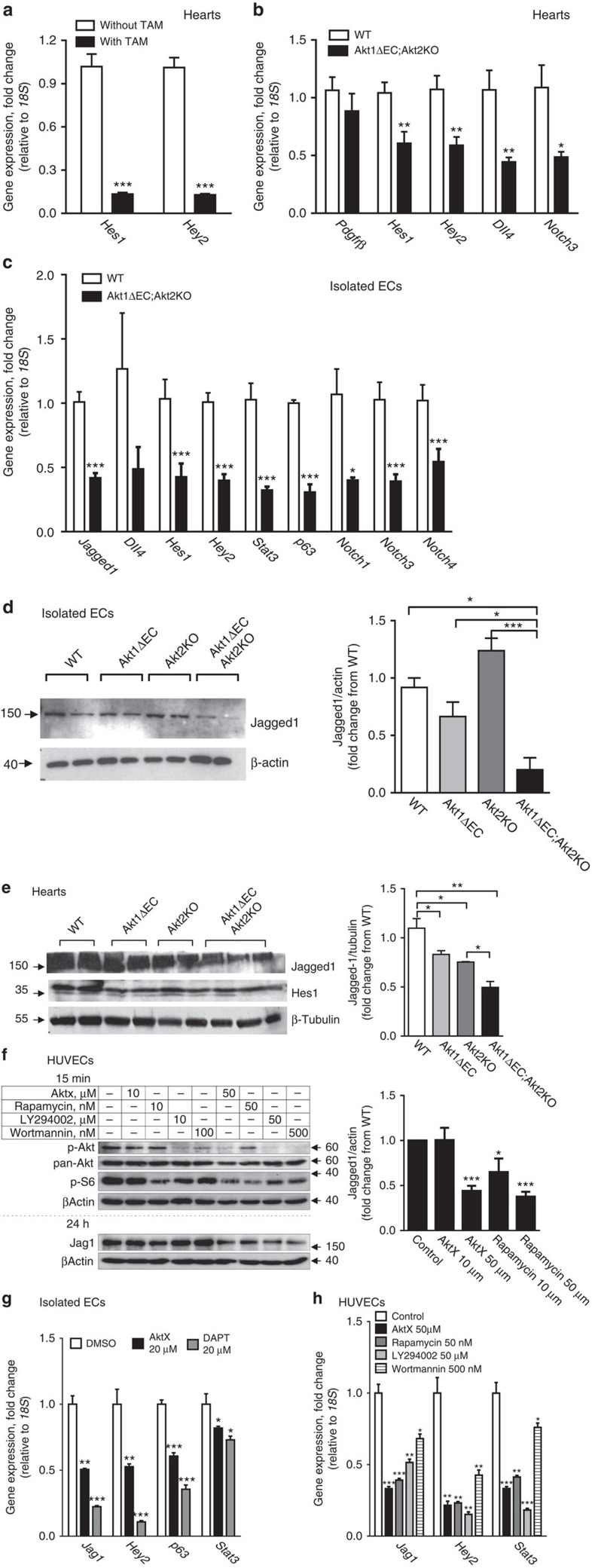
Notch signalling is diminished by interference with Akt/mTOR pathway. (**a**) Gene expression normalized to *18S* in Akt1ΔEC;Akt2KO hearts from littermates treated with and without tamoxifen (TAM) for 4 weeks represented as mean fold change from without TAM±s.e.m. (*n*=3–4). (**b**) Gene expression normalized to *18S* in WT and Akt1ΔEC;Akt2KO hearts after 4 weeks of tamoxifen treatment represented as mean fold change from WT±s.e.m. (*n*=3–4). (**c**) Gene expression normalized to *18S* in isolated ECs from WT and Akt1ΔEC;Akt2KO hearts after 4 weeks of tamoxifen treatment represented as mean fold change from WT±s.e.m. (*n*=3–4). (**d**,**e**) Representative immunoblotting and quantification of tamoxifen-treated isolated EC lysates after 3 days of 60 ng ml^−1^ VEGF treatment followed by 3 days of 1 μM tamoxifen treatment (**d**), and heart lysates from mice after 4 weeks of tamoxifen treatment (**e**) for actin and Jagged1 represented as mean±s.e.m. (*n*=2–3). (**f**) Representative immunoblotting and quantification of serum-starved HUVEC lysates after treatment with AktX, rapamycin, LY294002 or wortmannin for 15 min (p-Akt, pan-Akt and p-S6) or 24 h (p63 and Jag1) in the presence of 20% serum, 50 μg ml^−1^ endothelial growth supplement (ECGS) and 100 μg ml^−1^ heparin represented as mean±s.e.m. (*n*=2–3). (**g**) Gene expression normalized to *18S* in tamoxifen-treated isolated ECs after treatment with AktX or dual antiplatelet therapy (DAPT) for 24 h as mean fold change from dimethylsulfoxide (DMSO) control±s.e.m. (*n*=3–4). (**h**) Gene expression normalized to *18S* in serum-starved HUVECs after treatment with AktX, rapamycin, LY294002 or wortmannin in the presence of 20% serum, 50 μg ml^−1^ ECGS and 100 μg ml^−1^ heparin as mean fold change from control±s.e.m. (*n*=3). *P<0.05, **P<0.01 and ***P<0.005 by Student's *t* test (**a**–**c**) or one-way analysis of variance (**d**–**h**).

Since the main pathways controlling VSMC differentiation and recruitment and, possibly, retention involve either the Notch–Jagged1 or PDGFRβ axis, we analysed expression of related genes by quantitative RT–PCR in heart tissue from WT, Akt2KO, Akt1ΔEC and Akt1ΔEC;Akt2KO mice (Fig. 5). We documented that EC Akt1 excision triggered a marked reduction in Notch target expression in hearts (Fig. 5a,b). Indeed, tamoxifen-induced Akt1 deletion in Akt1ΔEC;Akt2KO mice caused >9-fold decrease in both *Hes1* and *Hey2* as evidenced by quantitative RT–PCR of whole hearts with and without tamoxifen treatment (Fig. 5a). Analysis of at least four hearts per genotype confirmed that expression of *Hes1*, *Hey2*, *Dll4* and *Notch3* in Akt1ΔEC;Akt2KO hearts was substantially lower than in WT hearts (Fig. 5b). The changes in *Pdgfrβ* (also a Notch target^41^) were substantially milder and did not reach statistical significance (Fig. 5b). This was further confirmed using primary EC isolated from Akt1ΔEC;Akt2KO mice. The analysis demonstrated >2-fold decrease in Notch signalling targets *Hes1*, *Hey2*, *Notch3*, *Notch1* and *Notch4* in ECs from Akt1ΔEC;Akt2KO mice compared with WT controls (Fig. 5c).

Since induced Akt1 deletion is endothelium-specific, it likely effects the endothelial components of Notch signalling, for example, Jagged1, which interact with Notch3 on VSMC to mediate their retention and differentiation from pericytes^42^,^43^,^44^,^45^,^46^. Since Jagged1 is expressed on several cell types, including VSMC, isolated EC were used. *Jagged1* gene expression was decreased >2-fold in ECs isolated from Akt1ΔEC;Akt2KO mice compared with WT ECs (Fig. 5c). This was translated into changes at the protein level of Jagged1 in isolated ECs, where Jagged1 level was dependent on the presence of Akt1 (Fig. 5d and Supplementary Fig. 16). Akt1 deletion but not Akt2 in ECs resulted in decreased Jagged1 levels compared with WT ECs. This expression was further diminished in ECs lacking both Akt isoforms (Akt1ΔEC;Akt2KO). As a result, Jagged1 levels in Akt1ΔEC;Akt2KO ECs were 4.6-fold lower than in WT ECs (Fig. 5d and Supplementary Fig. 16). Thus, in WT EC, the main isoform controlling Jagged1 expression is Akt1. However, in the absence of Akt1, this function can be performed by Akt2; therefore, there is an additive effect of Akt2KO on Jagged1 levels in Akt1-deficient but not WT EC.

Whole-heart immunoblotting confirmed that deletion of individual Akt isoforms resulted in reduction of Jagged1 *in vivo*, however, the most marked decrease was observed in Akt1ΔEC;Akt2KO hearts (Fig. 5e and Supplementary Fig. 16). As anticipated, this reduction in Jagged1 led to overall decreased Notch signalling as evidenced by mRNA and protein expression of main Notch targets in hearts (Fig. 5b,c,e). Results of immunoblotting show the gradual decrease in Hes1 levels in hearts with deletion of single Akt isoforms, and similar to Jagged1 expression, the most severe reduction was observed in Akt1ΔEC;Akt2KO hearts (Fig. 5e and Supplementary Fig. 16). Thus, the presence of Akt1 in EC may be essential for endothelial Jagged1 expression and the overall Notch signalling in the whole-organ context. Akt1 deletion in EC triggers a progressive decrease in Notch signalling in heart tissue, thereby diminishing VSMC differentiation, survival and vascular patency. The retinal vasculature development in inducible EC-specific Jagged1KO is characterized by reduced vascular branching and diminished VSMC coverage^47^. When Akt excision was induced under similar conditions (at P2), the phenotype of Akt1ΔEC;Akt2KO retinal vasculature showed similarities to EC-specific inducible Jagged1KO including reduced branching and lack of SMA staining (Supplementary Fig. 11 and Fig. 3f,g). The loss of VSMC in Akt1ΔEC;Akt2KO retinas was also reminiscent of *Notch3*^−/−^ mice^48^.

Although Akt has many downstream targets, Akt-mediated mTOR activation is one of the main determinants in transcriptional regulation. Thus, to assess the mechanism by which Akt regulates Jagged1 expression in endothelium, the components of this pathway were inhibited by AktX for Akt, rapamycin for mTOR, or LY294002 or wortmannin for PI3 kinase in human umbilical vein ECs (HUVECs). Interference with the PI3K/Akt/mTOR signalling pathway led to diminished pAkt and reduced mTOR activity based on p70 S6 levels (Fig. 5f and Supplementary Fig. 16). The blockade of Akt directly or by PI3K inhibitors diminished Jagged1 (Fig. 5f). Rapamycin treatment produced a similar effect demonstrating the involvement of mTOR in regulation of Jagged1 in ECs (Fig. 5f). Further, inhibition of Akt signalling by AktX or PI3K inhibitors diminished Jagged1 transcription in both HUVEC and mouse ECs as evidenced by changes in RNA levels (Fig. 5g,h and Supplementary Fig. 16). These results show that decreased Jagged1 is a direct consequence of interference with Akt/mTOR pathway in ECs. As expected, inhibition of this pathway led to markedly diminished expression of the main Notch target, *Hey2* (Fig. 5g,h), which, similar to *Jagged1*, was sensitive to the treatment with DAPT, a Notch pathway inhibitor (Fig. 5g). Similar effects on *Jagged1* and *Hey2* were achieved by inhibition of mTOR (Fig. 5h). During differentiation, transcriptional activity of mTOR pathway often involves p63 and STAT3 (refs 49, 50, 51). In endothelium, the blockade of Akt and mTOR resulted in reduced *p63* levels (Fig. 5g,h). Since STAT3 has been reported to serve as a transcriptional activator of p63 (ref. 52) and a downstream target of mTOR^49^,^53^, we examined *Stat3* expression on Akt inhibition in endothelium. The mRNA levels of STAT3 were decreased in cells treated with PI3K, Akt and mTOR inhibitors (Fig. 5g,h), suggesting that STAT3 might serve as a link connecting Akt/mTOR activation and p63 expression to induce Jagged1. In concert, gene expression of *Stat3* and *p63* were decreased in ECs isolated from Akt1ΔEC;Akt2KO mice compared with WT ECs (Fig. 5c). Thus, Akt controls Notch signalling by upregulating Jagged1 expression on EC via mTOR/STAT3/p63 pathway. Correspondingly, we assessed alterations in Notch downstream signalling in VSMCs isolated from Akt1ΔEC;Akt2KO and WT mice. In Akt1ΔEC;Akt2KO VSMCs, 65% of Notch signalling target genes expression was downregulated by more than 1.5-fold (Supplementary Table 1). These data demonstrate that EC Akt expression controls Jagged1 surface expression and Notch signalling in VSMCs.

### Jagged1 induction rescues VSMC loss and vascular patency

**Figure 6:**
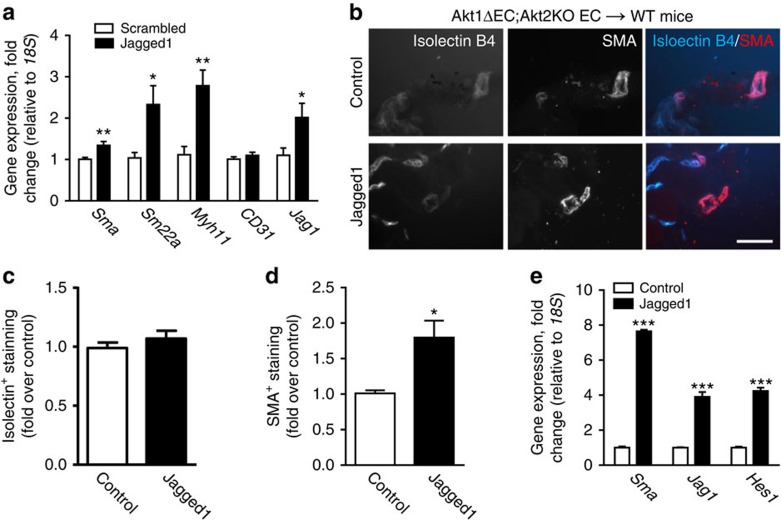
Jagged1 overexpression stimulates VSMC coverage. (**a**) Gene expression normalized to *18S* in Akt1ΔEC;Akt2KO hearts from littermates treated either 25 μg g^−1^ scrambled or Jagged1 peptide for 1 week during 2 weeks of tamoxifen treatment represented as mean fold change from scrambled±s.e.m. (*n*=6). (**b**) Representative images of sectioned matrigels containing control or Jagged1 retrovirus-infected Akt1ΔEC;Akt2KO ECs after injection into WT mice after 4 weeks tamoxifen treatment stained for isolectin B4 (blue) and SMA (red). Scale bar, 50 μm. (**c**,**d**) Quantification of isolectin B4^+^ and SMA^+^ staining intensity represented as mean fold intensity over control±s.e.m. (*n*=5–7). (**e**) Gene expression normalized to *18S* in matrigels containing control or Jagged1 retrovirus-infected Akt1ΔEC;Akt2KO ECs after injection into WT mice as mean fold change form control±s.e.m. (*n*=4–5). *P<0.05, **P<0.01 and ***P<0.005 by Student's *t*-test.

To demonstrate that VSMC loss in Akt1ΔEC;Akt2KO mice is caused by downregulation of Jagged1 we sought to correct the defects caused by Akt deficiency with Jagged1 peptide administration. Injections of Jagged1, but not scrambled control peptide increased VSMC marker expression (*Sma*, *Sm22a* and *Myh11*) in hearts of Akt1ΔEC;Akt2KO mice (Fig. 6a).

To further demonstrate that the rescue is EC-dependent, isolated EC from Akt1ΔEC;Akt2KO mice was treated with either control or Jagged1-encoding retrovirus. Jagged1-overexpressing or control Akt1ΔEC;Akt2KO ECs containing matrigels were injected subcutaneously in WT mice on tamoxifen and were sectioned to examine changes in vascular maturation (Fig. 6b). Quantification of isolectin^+^ cells demonstrated that an equal number of ECs was present in matrigels (Fig. 6c). Jagged1 overexpression in Akt1ΔEC;Akt2KO ECs resulted in a significant increase in the coverage of SMA^+^ VSMCs on the vasculature (Fig. 6d). In addition, Jagged1 overexpression, confirmed by gene expression of *Jagged1* and *Hes1*, increased *Sma* expression in matrigels (Fig. 6e). Thus, in two different experiments, Jagged1 rescued the deficiencies in VSMC retention caused by Akt shutdown. Importantly, Jagged1 re-expression in Akt-deficient ECs substantially improved vascular patency as indicated by the increased amount of blood in matrigel plugs containing Akt1ΔEC;Akt2KO ECs treated with Jagged1 retrovirus compared with Akt1ΔEC;Akt2KO ECs infected with control retrovirus (Supplementary Fig. 12). These data indicate that Akt regulation of Jagged1 levels on ECs is responsible for the maintenance of VSMC coverage of vasculature and, as a result, vascular patency.

### Endothelial Akt1 deletion causes organ dysfunction

**Figure 7:**
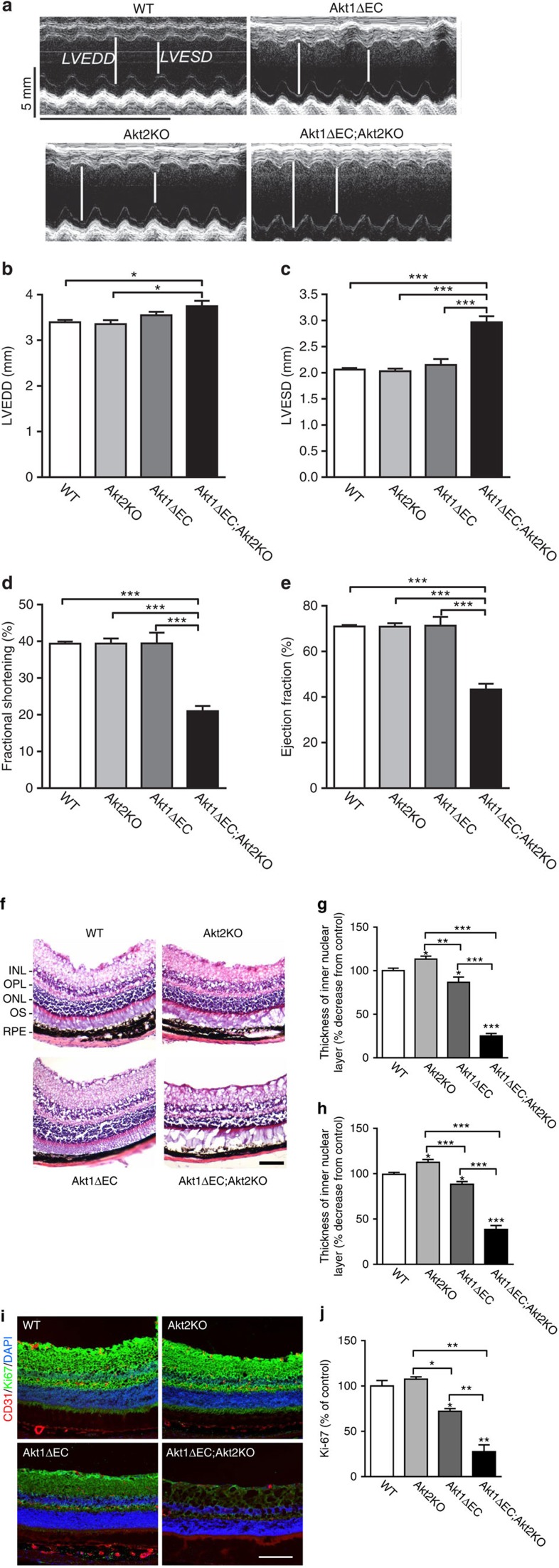
Functional tissue consequences from Akt ablation. (**a**) Representative echocardiography for WT, Akt2KO, Akt1ΔEC and Akt1ΔEC;Akt2KO mice after 4 weeks of tamoxifen treatment. Scale bar, 100 μm. (**b**–**e**) LVEDD, LVESD, percentage of fractional shortening and percentage of ejection fraction calculated from echocardiography representing mean±s.e.m. (*n*=4–6). (**f**) Representative images of WT, Akt2KO, Akt1ΔEC and Akt1ΔEC;Akt2KO retinas after tamoxifen treatment for 4 weeks stained with haematoxylin and eosin. INL, inner nuclear layer; ONL, outer nuclear area; OPL, outer plexiform layer; OS, inner and outer photoreceptor segment; RPE, retinal pigment epithelium layer and choroid. (**g**,**h**) Quantification of the thickness of the outer and inner nuclear layers represented as mean percentage decrease from control±s.e.m. (*n*=3–4). (**i**) Representative images of WT, Akt2KO, Akt1ΔEC and Akt1ΔEC;Akt2KO retinas after tamoxifen treatment for 4 weeks stained for CD31 (red), the proliferation marker Ki-67 (green) and DAPI (blue). Scale bar, 100 μm. (**j**) Quantification of the intensity of Ki-67 staining as mean percentage of control±s.e.m. (*n*=2). *P<0.05, **P<0.01 and ***P<0.005 by one-way analysis of variance.

Diminished Jagged1/Notch signalling in Akt null ECs and subsequent deterioration of vasculature led to severe organ dysfunction in Akt1ΔEC;Akt2KO mice. Echocardiography 4 weeks post initiation of Akt1 ablation revealed that EC-specific deletion of Akt1 in Akt2KO mice caused marked cardiac dysfunction (Fig. 7a–e) characterized by marked reduction in percentages of fractional shortening (Fig. 7d) and ejection fraction (Fig. 7e), whereas no substantial effect was observed in the absence of single isoforms (Fig. 7a–e). Correspondingly, Akt1ΔEC;Akt2KO hearts were dilated (Fig. 7b) and displayed increased heart/body weight ratio (Supplementary Fig. 13). The deleterious remodelling was observed only in Akt1ΔEC;Akt2KO but not in Akt2KO mice despite prolonged treatment with tamoxifen (10 weeks; Supplementary Fig. 14). Most marked cardiac dysfunction was observed in Akt1ΔEC;Akt2KO but not in Akt1ΔEC mice suggesting that in many tissues Akt2 isoform might compensate for the lack of Akt1. In the absence of Akt2, however, inducible deletion of Akt1 in endothelium, which is a minor cell type in heart tissue, causes severe cardiac dysfunction suggesting the requirement of Akt1/2 signalling in endothelial homeostasis.

Likewise, detrimental changes in blood supply to retina resulted in marked consequences. As shown in Fig. 7f, there was a substantial loss of cellularity of all layers in the retina in Akt1ΔEC;Akt2KO mice compared with WT and Akt2KO mice resulting in tissue regression and retinal dystrophy. Thickness of inner and outer nuclear retinal layers was decreased by 80% and 70%, respectively, in Akt1ΔEC;Akt2KO but not in Akt2KO mice compared with WT (Fig. 7g,h). Changes in single Akt1ΔEC mice were modest but still detectable (Fig. 7g,h). Vascular regression and tissue dystrophy were also evident in Akt1ΔEC;Akt2KO retinas stained for CD31, Ki-67 (to visualize proliferating cells) and 4,6-diamidino-2-phenylindole (DAPI) in comparison with WT and Akt2KO retinas (Fig. 7i). Cell proliferation was diminished by 77% and 30% in Akt1ΔEC;Akt2KO and Akt1ΔEC, respectively, compared with WT mice while no changes were observed in Akt2KO mice (Fig. 7j). Thus, loss of VSMC and vascular regression triggered by Akt shutdown in endothelium caused severe degeneration of retinas. The problems with eyes in Akt1ΔEC;Akt2KO but not in WT mice were associated with changes in size and position of the eye ball, inflammation and ulcerations, possibly due to scratching (Supplementary Fig. 15). Together, shutdown of endothelial Akt signalling causes gradual loss of VSMC differentiation markers and VSMC coverage followed by their apoptosis, severe vascular remodelling and regression of patent vasculature leading to tissue degeneration and organ failure.

## Discussion

The main findings are the following: (1) inducible endothelial Akt1 deletion on the Akt2KO background resulted in nearly complete ablation of Akt signalling in endothelium. Surprisingly, this did not affect EC survival, but led to a progressive loss of VSMC differentiation markers, and loss of arterial vasculature VSMC coverage followed by VSMC apoptosis at later time points. This led to structural changes in coronary vasculature, impaired vascular patency in the heart and severe cardiac dysfunction. (2) Likewise, Akt1 deletion in retinal endothelium resulted in the loss of VSMC and collagen IV coverage of blood vessels, vascular remodelling and vascular patency reduction leading to retinal dystrophy. (3) The main phenotypes of Akt KOs were recapitulated using Akt inhibitor *in vitro* and *in vivo*. (4) Analysis of gene expression in the absence of Akt1 *in vitro* and *in vivo* revealed marked decreases in Notch targets, including *Hes1* and *Hey2*, which was due to a diminished expression of a Notch ligand, Jagged1 on ECs. (5) Akt activity in adult endothelium is required for sustained Jagged1/Notch signalling and vascular stability and homeostasis as Jagged1 administration rescued VSMC loss *in vivo*.

Together, our study demonstrates that Akt's function in established vasculature is distinct from development. While global double Akt1/Akt2KO in mice is lethal^11^, ablation of both Akt1 and Akt2 in adult tissue did not cause massive cell death or apoptosis. Instead, inducible Akt KO reduced endothelial Jagged1 expression and perturbed endothelial interactions with VSMC, emphasizing that endothelium is not simply a barrier between blood and vessel wall, but a guardian of vasculature stability. This demonstrates that the function of Akt signalling in fully developed quiescent tissue is distinct from that during either developmental or postnatal vascular growth or remodelling. In developing vasculature, initial endothelial growth and sprouting is followed by Jagged1/Notch dependent pericyte differentiation into VSMC^45^, which cover and stabilize vessels further expanding arterial vasculature^44^,^48^. Cellular and molecular events triggered by Akt shutdown results in reduction of Jagged1/Notch signalling, loss of VSMC markers (de-differentiation) followed by their apoptosis, culminating in vascular thinning and regression, resembling a gradual reversal of vascular development. Under conditions of quiescence, the Akt pathway functions as a coordinator of heterologous cell–cell interactions and vascular stability.

Shutdown of Akt signalling in endothelium, a minor cell type in heart tissue compared with cardiomyocytes, fibroblasts and blood cells, resulted in marked vascular regression and severe organ dysfunction as early as 4 weeks after induction. This emphasizes a key role for endothelium in supporting patent vasculature, tissue perfusion and, as a result, performance of an organ with high energy and oxygen demands, such as the heart. Thus, nearly complete suppression of Akt signalling for prolonged time in endothelium alone might lead to vascular defects and insufficient tissue perfusion causing organ failure. Most potent Akt inhibitors demonstrated a similar extent of Akt inhibition in both the malignant and normal tissues of cancer patients (the median decrease was ∼89% based on pSer473)^35^. Administration of Akt inhibitor *in vivo* caused similar changes in VSMC recapitulating the phenotype of Akt KOs. Interestingly, various Akt inhibitors cause an unexpectedly wide range of adverse effects, ranging from skin rash to haematological complications, and importantly, including cardiac failure^35^,^37^,^38^,^54^. Altogether, this raises a question whether complete or nearly complete suppression of Akt signalling for a prolonged period of time will be safe in normal tissues, especially tissues exposed to the high concentrations of the drug, such as endothelium. At the very least, one can expect that inhibition of Akts in endothelium might lead to destabilization of existing vasculature, which, in turn, might affect neovascularization responses, such as wound healing or angiogenesis in tumours.

It remains unclear what upstream signalling is essential for this vascular stabilization function of Akts in endothelium. One could speculate that Akt activity in quiescent EC might be sustained, at least in part, by autocrine VEGF–VEGFR2 signalling in endothelium, which was essential for vascular homeostasis^40^. However, disruption of VEGF autocrine loop in endothelium led to endothelial apoptosis, microvascular infarctions and sudden death^40^, a phenotype clearly distinct from that observed in our study. Likewise, the direct involvement of another important upstream activator of Akt, PI3K signalling, in vascular stabilization is difficult to predict. Results of inducible ablation of PI3K signalling in vasculature are not available and ubiquitous KOs of selected PI3K isoforms exhibit rather severe phenotypes^30^,^31^,^32^, which are more complex and diverse that the one in our study. However, our finding that the lack of Akt signalling results in vascular destabilization can help to understand why neovascularization processes in ubiquitous Akt1 and PI3K KO mice as well as *in vitro* results might be so context dependent that they appear controversial^16^,^17^,^18^,^31^,^32^,^34^.

Our analysis revealed that deletion of Akt1 led to decreased expression of a Notch ligand and target, Jagged1, on ECs. Jagged1 on endothelium is required for induction of Notch3 in mural cells by an autoregulatory loop^43^,^55^. Increased Notch, in turn, increases Jagged1 expression thereby resulting in amplification of the signal^56^,^57^. Thus, reduction in Jagged1 might be sufficient to cause diminished Notch signalling in the context of the entire heart. Indeed, we show that deletion of Akt1 in EC of Akt1ΔEC;Akt2KO mice led to marked changes in the expression of the main Notch targets, exemplified by *Hes1*, *Hey2* and *Dll4*, in whole-heart tissue. Notch signalling is a well-established regulator of vascular maturation and arteriogenesis during development^42^,^44^,^56^. Expression of Notch targets^58^, especially *Hey2*, is essential not only for coronary vascular maturation during development^59^ but also for overall cardiac development and function^58^. Despite the overwhelming evidence that Jagged1/Notch signalling is a master regulator of mural cell differentiation and vascular maturation in development, little is known regarding the consequences of Notch inhibition in adults. The role of Notch was shown in the context of either neoangiogenesis^44^,^60^,^61^,^62^,^63^ or remodelling^63^ processes triggered by injury^63^,^64^ rather than in the vascular stability and homeostasis. Literature analysis^60^,^65^,^66^ allows speculation that interference with Jagged1/Notch signalling in adult vasculature will lead to substantial vascular destabilization similar to inducible Akt KOs. Observed reduction in Dll4 combined with diminished Jagged 1 in Akt1ΔEC;Akt2KO mice might further augment the observed defects in VSMC coverage as suggested by a recent study^63^. More direct comparison of the consequences of Akt EC KOs to that of Jagged1 EC KO^47^,^63^ in retinal model revealed a number of similarities, including reduced branching and, most importantly, severe lack of VSMC coverage. However, the phenotype of EC-specific Jagged1KO is considerably milder than one of its upstream regulator, Akt, due to the existence of other, possibly interdependent pathways of vascular maturation, including Angiopoietin2 as recently described by Ju *et al.*^67^ Likewise, KOs of signalling molecules upstream of the same pathway may display more severe phenotypes. Indeed, the defects in vascular maturation during angiogenesis in mice expressing inactive form of p110alpha isoform of PI3K (ref. 32) are even more pronounced than in Akt KO. However, it is possible that the observed phenotype with expression of inactive PI3K including increased vascular density and reduced pericyte coverage could be due to alterations in Jagged1 downstream of Akt activation.

Together, it is possible that imbalance of Jagged1/Notch signalling resulting from Akt inhibition might be a contributing factor to hyper branching and immature vascular phenotypes observed in neovascularization models exemplified by tumour angiogenesis^17^,^32^,^68^. Thus, our results demonstrate that endothelial Akt pathway in fully developed vasculature is essential for the maintenance of Notch ligand expression, subsequent Notch signalling and vascular stability.

In sum, we show that a complete shutdown of Akt signalling in endothelium of adult mice has unexpected yet detrimental consequences on mature vasculature in several tissues and cardiac function suggesting the essential role for this pathway in vascular homeostasis.

## Methods

### Experimental animals

Mice with tamoxifen-inducible Cre recombinase expression under the VE-Cadherin promoter (Cdh5(PAC)-Cre^ERT2^) on a C57BL/6 background were generated by Ralf H. Adams^69^. These mice were crossed with Akt1^fl/fl^ mice (generated by Dr Nissim Hay; C57BL/6 background)^70^ to generate Cdh5(PAC)-Cre^ERT2^;Akt1^fl/fl^ (Akt1ΔEC). These mice were then crossed with Akt2^−/−^ (Akt2KO; from Dr Nissim Hay; C57BL/6 background) mice to generate Cdh5(PAC)-Cre^ERT2^;Akt1^fl/fl^;Akt2^−/−^ (Akt1ΔEC;Akt2KO). C57BL/6 (WT) mice were used as controls. To generate reporter mice, Cdh5(PAC)-Cre^ERT2^;Akt1^fl/fl^;Akt2^−/−^ (Akt1ΔEC;Akt2KO) were crossed with mT/mG reporter mice (from Jackson Labs). In the resultant mT/mG;Cdh5(PAC)-Cre^ERT2^;Akt1^fl/fl^;Akt2^−/−^ (mT/mG;Akt1ΔEC;Akt2KO) mice, all tissues express the red membrane targeted (m)-tomato. However, in tamoxifen-treated mT/mG;Akt1ΔEC;Akt2KO mice, where the Cre recombinase gene is expressed, the ECs express the membrane targeted green fluorescent protein (m-GFP) on their surface. Thus, the Cre recombinase expression induced by tamoxifen can be easily tracked in these mice indicating where the Akt1 gene has been deleted. Age- and gender-matched mice were used for all experiments. No differences were noted between male and female mice in cardiac function or heart weight to body weight ratios. Mice were subdivided into experimental groups based on the tissues being collected and interventions performed. All animal procedures were performed in accordance with the Institutional Animal Care and Use Committee of the Cleveland Clinic guidelines. Experiments were terminated, at the latest, 10 weeks after tamoxifen treatment as requested by the veterinarian due to inflammation and ulcerations developing on the eyes of Akt1ΔEC;Akt2KO mice.

### Tamoxifen treatment

For intraperitoneal (i.p.) injections, tamoxifen (Sigma) was dissolved in 100% ethanol and then diluted in corn oil to a final concentration of 20 mg ml^−1^. Four- or five-week-old mice were injected daily with 4 μl per g body weight for 5 days. After injection, mice were placed on tamoxifen diet (Harlan Tekland) until experimental termination (either 3 or 9 additional weeks) resulting in a total of 4 or 10 weeks of tamoxifen treatment and mice aged 8–9 weeks after 4 weeks of tamoxifen or 14–15 weeks after 10 weeks of tamoxifen treatment. Continuous tamoxifen diet after injections resulted in a greater decrease in total Akt as judged by immunoblotting, especially at the later time points (10 weeks after initiation of excision). This regimen of tamoxifen treatment resulted in successful excision of Akt1 in genomic DNA as demonstrated by the presence of the ∼300-bp band (Supplementary Fig. 1). This treatment schedule was chosen based on monitoring the total Akt levels in tissue lysates (heart, spleen and liver as shown in Supplementary Figs 2 and 3). We did not observe a substantial effect of tamoxifen on vascular parameters in either WT, Akt2KO or Cre(−)Akt1ΔEC;Akt2KO mice. In all experiments, Akt2KO and Cre(−)Akt1ΔEC;Akt2KO exhibited similar phenotypes, therefore, either of these controls are shown. For cell culture experiments, treatment with 60 ng ml^−1^ VEGF (R&D Systems) was used to induce VE-Cadherin expression in adult murine lung ECs. After 3 days, 1 μM 4-hydroxytamoxifen (Sigma) was added to cultures to induce genetic deletion of Akt1 in isolated ECs for 3 days. Cultures were then used in subsequent experiments.

### Tamoxifen regimen rationale

The *in vivo* tamoxifen regimen was optimized for maximal deletion of Akt1 in the ECs of adult tissues. The 5 days of i.p. injection was found to be minimally sufficient to induce excision of Akt1 in genomic DNA with minimal impact on the health of the animals. After the initial injections, the concern was that the excision in adult tissues could be mosaic, as *Cdh5* expression is decreased after development in most tissues. This mosaic excision would result in endothelium containing both WT and Akt1ΔEC cells, and one cell type could have a growth advantage leading to a selection of one cell type over the other. To prevent this from occurring, we maintained mice on a tamoxifen diet throughout experimental manipulation to ensure continued deletion of Akt1 in adult endothelium. This tamoxifen regimen of 5 days i.p. injection followed by at least 3 weeks of tamoxifen diet (4 weeks of tamoxifen treatment in total) was shown to efficiently reduce Akt1 expression in tissues, with hearts displaying the highest decrease in Akt1 levels (Supplementary Fig. 3).

To determine that the EC Akt1 deletion was the main cause of all phenotypic changes, we utilized two experimental approaches with controls. First, we used separate murine genotypes: WT, Akt1ΔEC, Akt2KO and Akt1ΔEC;Akt2KO mice, all treated with 4 weeks of tamoxifen to account for any side effects of tamoxifen treatment. As the excision does not occur in WT or Akt2KO mice, any changes seen in these mice would be a side effect of tamoxifen. Second, we used Akt1ΔEC;Akt2KO littermates separated into groups: one with and one without 4 weeks of tamoxifen treatment. This experimental approach allowed us to directly compare the effects of EC Akt1 deletion due to tamoxifen in the same genotype using age- and gender-matched littermates.

The *in vitro* tamoxifen regimen was again based on a loss of *Cdh5* expression in adult tissues. Lung tissues from mice after 4 weeks of tamoxifen treatment showed a very small decrease in Akt1. Thus, we treated all isolated lung ECs with 60 ng ml^−1^ VEGF for 3 days to induce *Cdh5* expression. Then, cells were treated with 1 μM tamoxifen for 3 days to induce deletion of Akt1, which was confirmed by immunoblotting (Supplementary Fig. 2).

### KOs of Akts in endothelium

To visualize Cre recombinase expression in adult tissues, Cdh5(PAC)-Cre^ERT2^;Akt1^fl/fl^;Akt2^−/−^ (Akt1ΔEC;Akt2KO) were crossed with mT/mG reporter mice. The highest levels of tamoxifen-induced Cre recombinase activity were found in heart and retinal tissues (Supplementary Fig. 2A). The percentage of mG^+^ and CD31^+^ ECs was >90% in heart and >80% in retinas (Supplementary Fig. 2B). In ECs isolated directly from tamoxifen-treated hearts, gene expression of *Akt1* was decreased >95% (Supplementary Fig. 2C). In isolated lung ECs tamoxifen decreased total Akt levels by >35% in Akt1ΔEC cells and by >80% in Akt1ΔEC;Akt2KO cells (Supplementary Fig. 2D,E). In most organs, EC represent a minor cell type (often <5% of all cells), thus, deletion of endothelial Akt1 alone is not expected to markedly affect total Akt levels in whole-organ lysates (Supplementary Fig. 3). Immunostaining of matrigel plugs for CD31 and Akt1 demonstrated that ECs in Akt1ΔEC;Akt2KO mice do not express Akt1, while the blood cells retain Akt1 (Supplementary Fig. 2F). However, during neovascularization in matrigel plugs, ECs represent ∼50% of the total cell content (Supplementary Fig. 2G), whereas the other 50% are blood and mural cells. Gene expression analysis revealed that EC Akt1 deletion in Akt1ΔEC;Akt2KO mice resulted in ∼33% reduction of total *Akt1* (Supplementary Fig. 2H), since matrigels are an equal mixture of ECs and non-ECs (Supplementary Fig. 2G) this likely represents a complete deletion of *Akt1* in ECs. *Akt2* was below the detection limit (Supplementary Fig. 2H). Thus, excision of Akt1 in ECs *in vivo* was complete. The changes in Akt levels were detectable in whole-heart lysates where Akt deletion led to ∼40% decrease in Akt1 and 40% and 30% reduction in phospho-Akt (T308) and pan-Akt, respectively, in Akt1ΔEC;Akt2KO mice compared with WT mice, whereas Akt2 was absent (Supplementary Fig. 3C). Importantly, no compensatory changes in the levels of remaining Akt isoform, Akt3 were detected (Supplementary Fig. 3).

### Endothelial cell isolation

Lungs were isolated and digested in 3 mg ml^−1^ collagenase–dispase mixture for 4 h. Digested tissues were strained and recovered murine lung endothelial cells were plated on 10 μg ml^−1^ fibronectin in DMEM supplemented with 10% FBS, 90 μg ml^−1^ heparin sulfate and 50 ng ml^−1^ endothelial cell growth supplement. Once confluent, murine lung endothelial cells were purified by positive selection using anti-CD31 (BD Pharmigen)-coated Dynal beads (Invitrogen) and used in subsequent experiments after tamoxifen treatment.

Hearts were isolated and digested in 3 mg ml^−1^ collagenase–dispase mixture for 4 h. Digested tissues were strained and recovered ECs were purified by positive selection using anti-CD31 (BD Pharmigen)-coated Dynal beads (Invitrogen) and used in subsequent experiments directly after isolation.

### Immunoblotting

ECs were lysed in RIPA buffer containing 1 mM protease inhibitors. Hearts were homogenized and lysed in NP-40 lysis buffer containing aprotinin and leupeptin hemisulfate salt (Sigma). Samples were separated by electrophoresis and transferred to a polyvinylidene difluoride membrane. Proteins were detected with (1:1,000) antibodies against Akt1, Jagged1, Hes1, GADPH (all from Santa Cruz Biotechnology), Akt2, Akt3, pan-Akt, phospho-Akt(S473), phospho-Akt(T308), phospho-S6 (all from Cell Signalling Technologies), p63 (from Genetex), tubulin and β-actin (all from Sigma). Densitometry was performed using NIH ImageJ. Full scans of western blots are presented in Supplementary Fig. 16.

### Matrigel plug assay

Matrigel (500 μl; BD Biosciences) containing VEGF (250 ng) was injected subcutaneously (s.c.) into tamoxifen-treated mice during the last week of diet. Plugs were removed after 7 days and processed for immunofluoresence or used in quantitative RT–PCR analysis. For experiments with Jagged1 re-expression, ECs were infected with a retrovirus expressing Jagged1 or a control retrovirus. In all, 5 × 10^5^ infected ECs were encapsulated in matrigel containing VEGF and injected s.c. into tamoxifen-treated WT mice. Plugs were removed after 14 days and processed for immunofluorescence or used in quantitative RT–PCR analysis.

### Immunofluorescence

Hearts, eyes or matrigels were embedded in OCT freezing medium and sectioned at 7 μm. Sections were then fixed in 4% paraformaldehyde (PFA) and incubated with primary antibodies raised against CD31 (1:50, BD Pharmigen), GFP (1:200, Rockland), Akt1 (1:100, Cell Signaling), isolectinB4-biotin (1:50, Life Technologies), Ki-67 (1:50, Dako), cleaved caspase 3 (1:50, Cell Signaling), Jagged1 (1:50, SantaCruz), LYVE1 (1:50, Abcam), Myh11 (1:50, Abcam), NG2 (1:50, Millipore) or anti-SMA (1:100, Abcam). The Akt1 antibody was tested for specificity on global Akt1KO tissues and did not show nonspecific staining. Tissues were then washed in PBS and exposed to a fluorescently labelled secondary antibodies (Invitrogen). In addition, antibodies directly conjugated to fluorophores were used including wheat germ agglutinin-488 (Life Technologies) and SMA-Cy3 (Sigma). Slides were mounted with Vectamount+DAPI (Vector Labs) and images were taken using either a TCS-SP (Leica) or a ZMZ1000 (Nikon) microscope. The images were quantified using ImagePro software (Media Cybernetics).

For cleaved caspase 3 quantification, the estimated area of smooth muscle surrounding each vessel (∼20 μm from the wall of the vessel gauged by shadow in background staining) was traced, followed by using the ‘clear outside' function. Following this, colour channels were split and for each channel, threshold values were set and area measurements were taken. Area for each vessels cleaved caspase 3 staining was divided by CD31 staining area. Final percentages for vessels from each heart were then averaged.

### Quantitative PCR

For real-time RT–PCR, total RNA from whole eyes, hearts and implanted matrigel plugs were extracted using the RNeasy Micro kit (QIAGEN, Valencia, CA), according to the manufacturer's instructions. Primer sequences can be found in Supplementary Table 2. The RNA was converted into cDNA using the Omniscript kit (QIAGEN). *18S* mRNA served as a control for relative quantification. For detection and quantification, a MyiQ real-time PCR detection system (Bio-Rad Laboratories) was used. PCR reactions were performed using an iQ SYBR Green Supermix kit (Bio-Rad Laboratories). Data was analysed by the ΔΔC_T_ method.

### Microcomputed tomography

After 10 weeks of tamoxifen treatment, mice were anesthetized (100 mg ketamine per kg body weight and 10 mg xylazine per kg body weight) and injected with 500 U ml^−1^ heparin followed by saturated potassium chloride solution. The heart was exposed, catheterized and flushed with saline, followed by a vasodilator (saline with 1,000 U l^−1^ heparin, 4 mg ml^−1^ papaverine and 1 g l^−1^ adenosine) and 2% PFA. A 20% bismuth nanoparticle in 5% gelatin was injected as a contrast agent. Hearts were removed and fixed in 2% PFA. Tissues from mice were excluded from analysis if the tissue structure was compromised either due to ruptures of the major blood vessels or a lack of sufficient perfusion. The vasculature in the heart was scanned with a high-resolution microCT imaging system (GE eXplore Locus SP, GE Healthcare) with a cone beam-filtered back projection algorithm, set to a 0.008-mm effective detector pixel size. The microCT was operated with 60-kVp X-ray tube voltage, 100-mA tube current, 2,960-ms per frame, 1 × 1 detector-binning model, 360°, and 0.5° increments per view. This acquisition resulted in a set of contiguous VFF-formatted images through the entire heart. Microview Software (GE Healthcare) was used to correct and initially reconstruct raw data with voxels of dimensions 16 μm × 16 μm × 16 μm for visualization. Then this reconstructed microCT data set was transferred to the Advanced Workstation (version 4.4; GE Healthcare), different post-processing techniques were used to process segmentation (separate the arterial system from the contaminating venous system) and re-batch the reconstructed arterial system according to the re-orientated central line along the long axis of the left ventricle using a modified method for quantification. Re-orientation is a critical step for minimizing the quantitative error (change in the cross-sectional shape of the vascular tree from oval to circle). Multiplanar reformatting techniques permitted us to view the data set in transverse, sagittal, coronal and hybrid planes. Detailed morphometric data on the diameters, area, number of vessels and distributions of different sized vessels were extracted using modified software (ImageJ; National Institutes of Health, Bethesda, MD). Afterwards, distribution of the relative vessel number was automatically calculated with the built-in algorithm. The data were expressed as a vascular segmental number, representing the total number of vessels, of a specified diameter, counted in total reformatted cross-sections for the whole heart.

### Lectin perfusion

To visualize blood vessels in hearts and retinas, we used lectin perfusion. Four- to five-week-old littermates were separated into two groups: one with and one without tamoxifen treatment for 4 weeks. The now 8- to 9-week-old littermates were anesthetized (100 mg ketamine per kg body weight and 10 mg xylazine per kg body weight) and injected intravenously with 100 μg of FITC-labelled lectin (*Lycopersicon esculentum*; Vector Laboratories). After 15 min, hearts were removed and frozen in OCT medium and sectioned at 30 μm. Meanwhile, retina were collected and fixed with 4% PFA at 4 °C overnight. Lectin staining was visualized using Apollo confocal microscope (Leica). The 3D image visualization and quantification were performed by Volocity software.

### Retina isolation and whole-mount staining

Eyes were isolated from adult mice treated with 4 or 10 weeks of tamoxifen as described or from 10-day-old pups that were fed tamoxifen from P2 to P6. Eyes were fixed in 4% PFA and then the retinas dissected out. For isolectin B4 staining, retinas were permeabilized in 0.5% Triton X-100/1% BSA/PBS for 2 h. For all staining, retinas were incubated with primary antibodies anti-CD31 (1:50), anti-collagen type IV (1:100, Abcam), anti-GFP (1:200), anti-SMA (1:100) or isolectin B4 (1:50) followed by secondary antibodies at 1:1,000 dilution. Staining was visualized using confocal microscopy (Leica TSC SP2).

### HUVEC treatment

HUVEC cells (a kind gift from Dr DiCorleto) were serum starved and then treated with 10 or 50 μM AktX (Calbiochem), 10 or 50 nM rapamycin (LC Laboratories), 10 or 50 μM LY294002 (Cell Signaling Technologies), 20uM DAPT (N-[(3,5-Difluorophenyl)acetyl]-L-alanyl-2-phenyl]glycine-1,1-dimethyl ester or 100 or 500 nM Wortmannin (Sigma-Aldrich) inhibitors for 15 min or 24 h. Cells were then lysed for immunoblotting or RNA was isolated using the Qiagen RNeasy kit.

### Jagged1 peptide treatment

Mice at 21 days of age were treated for 5 days of tamoxifen i.p. followed by 3 weeks of tamoxifen diet. During the last 2 weeks, mice were injected s.c. daily with 25 μg g^−1^ Jagged1 (CDDYYYGFGCNKFCRPR) or scrambled (RCGPDCFDNYGRYKYCF) peptide (New England Peptides). Mice were then killed and heart and eyes were collected for quantitative RT–PCR.

### Echocardiography

Cardiac function was assessed in mice after 4 weeks of tamoxifen diet (∼8–9 weeks of age). *In vivo* heart function was assessed using a Vivid 7 ultrasound machine (GE Medical) equipped an il3L linear probe operated at 14 MHz. Mice were imaged in a conscious state at a room temperature of 73 °F and with decreased ambient lighting while held by an experienced handler in a supine left decubitus position. Mice were placed on an adjustable platform equipped with echocardiograph electrodes to monitor heart and respiration rates. The heart was imaged in the two-dimensional mode in the parasternal long and short-axis views with a depth setting of 1.0 cm and at a frame rate of ≥275 frames per s. Left ventricular (LV) area was measured from short-axis views at papillary muscle levels, and an M-mode image was obtained at a sweep speed of 200 mm s^−1^. Diastolic LV wall thickness, systolic LV wall thickness, LV end-diastolic dimension (LVEDD), and LV end-systolic chamber dimension (LVESD) were measured. All measurements were done from leading edge to leading edge according to American Society of Echocardiography guidelines. The percentage of LV fractional shortening (FS) was calculated as follows: FS=(LVEDD−LVESD)/LVEDD.

### BBB permeability

BBB permeability was assessed using sodium-fluorescein (NaF) (Sigma), a low-molecular mass molecule (376 Da), to detect fluid-phase shifts between the circulation and central nervous system. Briefly, mice received 100 μl of 10% NaF in PBS intraperitoneally and cardiac blood was collected 10 min later. Mice were transcardially perfused with 10 ml of PBS, and spinal cord and brain removed. Each tissue was weighed, homogenized in PBS using a TissueLyser (Qiagen) and centrifuged at 14,000*g* for 2 min. A volume of 500 μl of clarified supernatant was added to 500 μl of 15% trichloroacetic acid (Sigma) and centrifuged at 10,000*g* for 10 min. A volume of 125 μl of 5 N NaOH (Sigma-Aldrich) was added to 500 μl of the supernatant and NaF content measured on a SpectraMax Mz (Molecular Devices) microplate reader using standards ranging from 125 to 4,000 μg. NaF in spinal cord or brain was normalized to sera NaF content using the formula: (mg fluorescence brain tissue per mg of protein)/(mg fluorescence sera per μl of blood). Data are expressed as fold increases in NaF in the spinal cord or brain with levels from WT-treated mice set at 1.

### VSMC isolation

Smooth muscle cells were isolated from aortic outgrowths. Aortae will be collected from mice, cleaned and the intimal layer removed. The aortae were then minced and placed into dishes. After ∼2 weeks, smooth muscle cells had migrated onto the dish and aortic explants were removed. Smooth muscle cells were further subcultured and used in the following experiments.

### Quantitative PCR arrays

Total RNA was isolated from smooth muscle cells using the Qiagen RNeasy kit and cDNA was synthesized using the Qiagen RT^2^ First Strand kit. Real-time PCR was performed using Qiagen RT^2^ Profiler PCR Array kit for Mouse Notch Signaling Targets and measured on a Bio-Rad myIQ2 iCycler. Data were analysed by the ΔΔC_T_ method using the SABiosciences PCR Array Data analysis tools to calculate fold change increased expression or decreased expression in Akt1ΔEC samples compared with WT.

### Akt inhibition

Mice at 21 days of age were treated orally three times per week with 120 mg kg^−1^ MK2206 (SantaCruz Biotech) in 30% capitsol (Cydex Pharmaceuticals) for 2 weeks. Mice were then killed and heart and eyes were collected for quantitative RT–PCR.

### Statistical analysis

Student's *t*-test analysis or one-way analysis of variance analysis with Newman–Keuls post-test were used to determine statistical significance using GraphPad Prism 5.0 software. Error bars represent the s.e.m. of experiments. *P<0.05, **P<0.01 and ***P<0.005. Experiments were repeated at least three times with 4–6 mice per genotype.

## Additional information

**How to cite this article:** Kerr, B. A. *et al.* Stability and function of adult vasculature is sustained by Akt/Jagged1 signalling axis in endothelium. *Nat. Commun.* 7:10960 doi: 10.1038/ncomms10960 (2016).

## Supplementary Material

Supplementary Information Supplementary Figures 1-16 and Supplementary Tables 1-2

Supplementary Movie 1 Perfusion of AktΔEC;Akt2KO heart without Akt1 excision. Representative images of AktΔEC;Akt2KO hearts of littermate mice perfused with lectin without tamoxifen (TAM) treatment.

Supplementary Movie 2 Perfusion of AktΔEC;Akt2KO heart after Akt1 excision. Representative images of AktΔEC;Akt2KO hearts of littermate mice perfused with lectin with tamoxifen (TAM) treatment for 4 weeks (5 days i.p. tamoxifen injection followed by 3 weeks tamoxifen diet).

Supplementary Movie 3 Perfusion of AktΔEC;Akt2KO retina without Akt1 excision. Representative images of AktΔEC;Akt2KO retinas of littermate mice (8-9 weeks of age) perfused without tamoxifen (TAM) treatment.

Supplementary Movie 4 Perfusion of AktΔEC;Akt2KO retina after Akt1 excision. Representative images of AktΔEC;Akt2KO retinas of littermate mice (8-9 weeks of age) perfused with lectin with tamoxifen (TAM) treatment for 4 weeks (5 days i.p. tamoxifen injection followed by 3 weeks tamoxifen diet).

Supplementary Movie 5 Retinal Akt2KO vessel regression after tamoxifen treatment. Representative images of Akt2KO whole mount retinas (8-9 week old mice) after 4 weeks of tamoxifen treatment (5 days i.p. tamoxifen injection followed by 3 weeks tamoxifen diet) stained for collagen IV (red) and isolectin B4 (green).

Supplementary Movie 6 Retinal AktΔEC;Akt2KO vessel regression after Akt1 excision. Representative images of AktΔEC;Akt2KO whole mount retinas (8-9 week old mice) after 4 weeks of tamoxifen treatment (5 days i.p. tamoxifen injection followed by 3 weeks tamoxifen diet) stained for collagen IV (red) and isolectin B4 (green).
