# Supplementary material for: Stability and function of adult vasculature is sustained by Akt/Jagged1 signalling axis in endothelium
Source: Nat Commun. 2016 Mar 14;7:10960. doi: 10.1038/ncomms10960 (PMC4793084; doi:10.1038/ncomms10960)
Supplement: Supplementary Information — Supplementary Figures 1-16 and Supplementary Tables 1-2 [file ncomms10960-s1.pdf]

Supplementary Figure 1

A

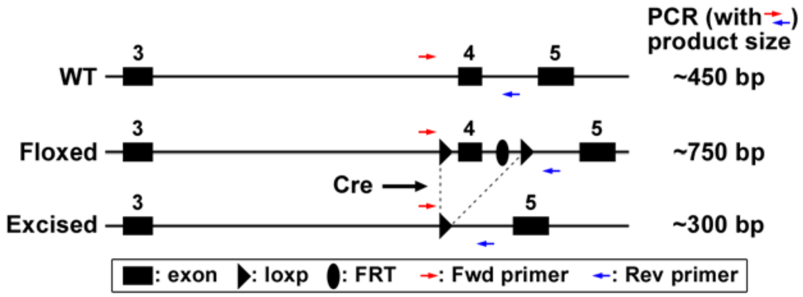

B

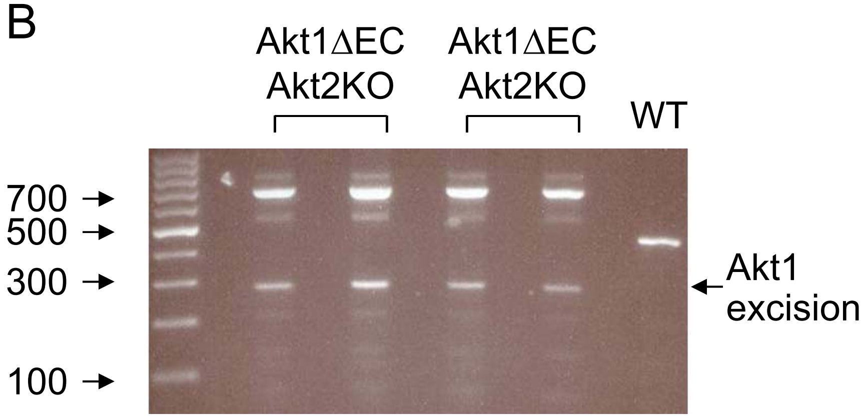

Supplementary Figure 1. Akt1 excision in genomic tail DNA. (A) Experimental design for PCR amplification of mouse tail genomic DNA from Akt1ΔEC; Akt2KO, and WT mice. The forward (in red) and reverse (in blue) PCR primers are flanking the exon 4 region of Akt1 genomic DNA and their sequences are TCACAGAGATCCACCTGTGC and GGGCCTCCATACACTCAAGA, respectively. The ~450 bp band corresponds to the WT allele without loxp insertion, ~750 bp band to the floxed Akt1 allele before Cre-mediated excision (in all tissues of Akt1ΔEC; Akt2KO mice), and ~300 bp band to the excised allele (from endothelial cells of Akt1ΔEC; Akt2KO mice). (B) Results of PCR amplification of mouse tail genomic DNA from Akt1ΔEC;Akt2KO (representative samples from 2 mice in duplicate) and WT mice after 5 days of i.p. injections of 4μL/g body weight tamoxifen (20mg/mL) followed by tamoxifen diet for 3 weeks (4 weeks of tamoxifen treatment). The 450 bp band corresponds to the WT allele and the ~300bp band to the excised Akt1 allele.

Supplementary Figure 2

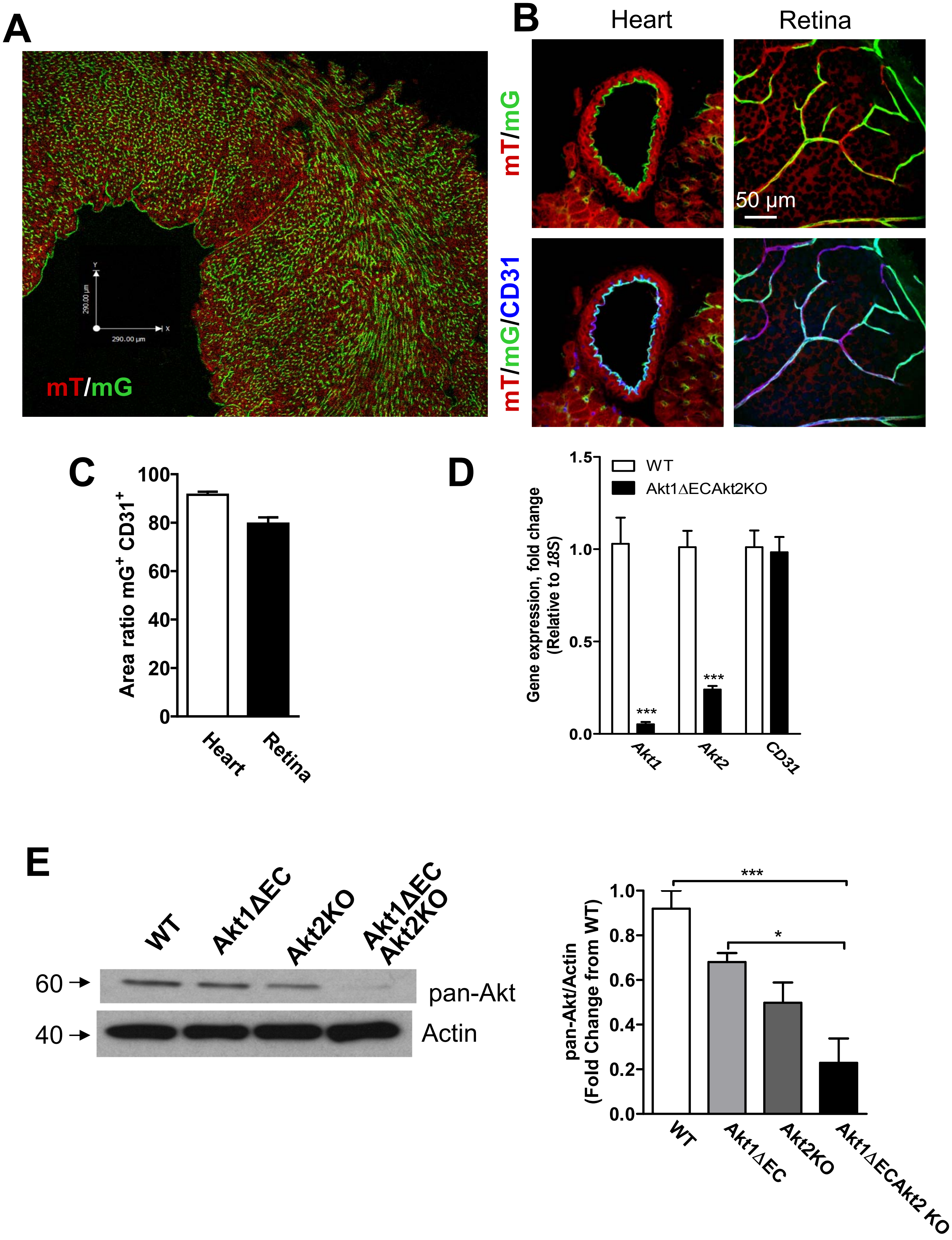

F

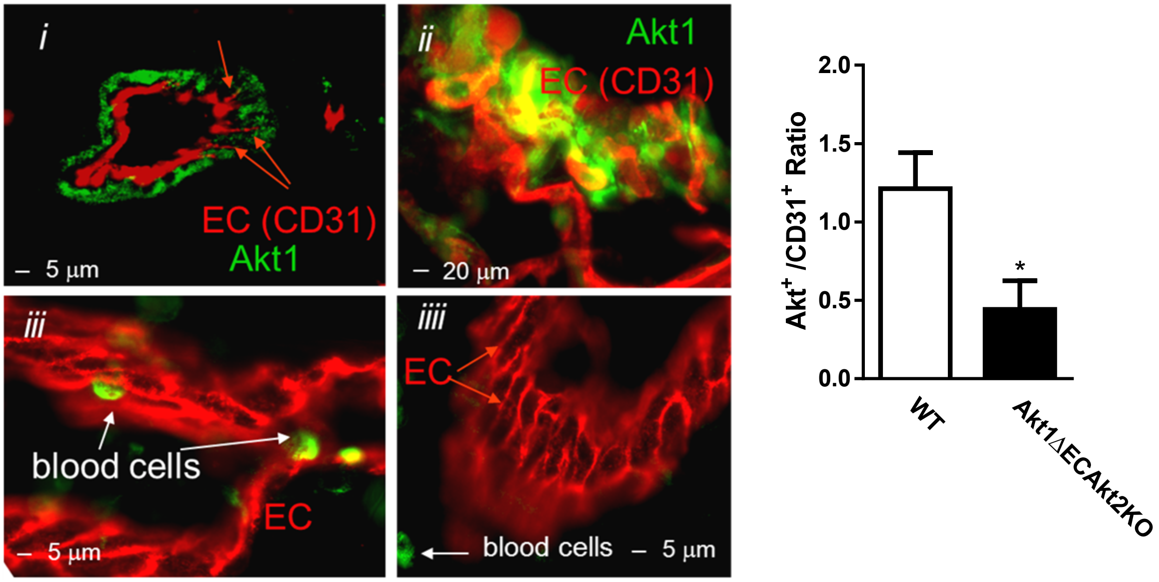

i
EC (CD31)
Akt1
5 μm
ii
Akt1
EC (CD31)
20 μm
iii
blood cells
EC
5 μm
iiii
EC
blood cells
5 μm
2.0
1.5
1.0
0.5
0.0
Akt+ /CD31+ Ratio
*
WT
Akt1ΔECAkt2KO

G

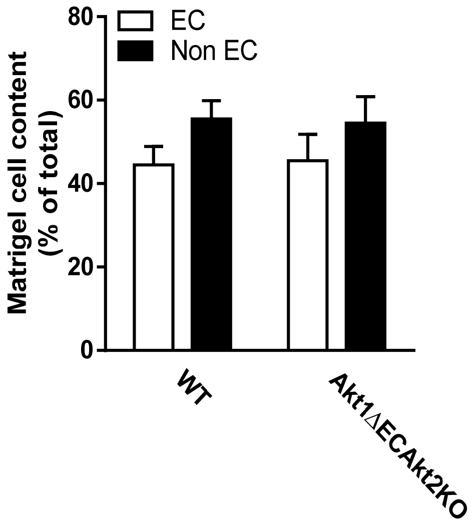

80
60
40
20
0
Matrigel cell content
(% of total)
EC
Non EC
WT
Akt1ΔECAkt2KO

H

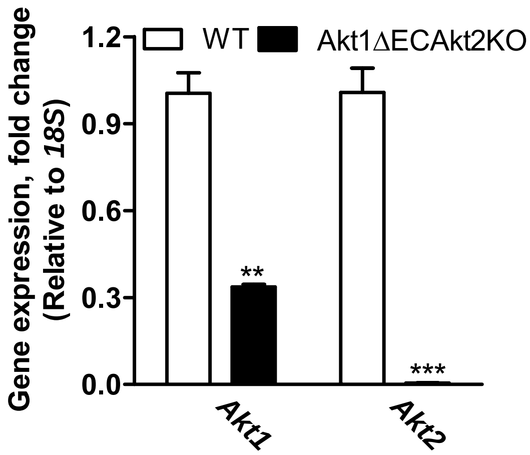

1.2
0.9
0.6
0.3
0.0
Gene expression, fold change
(Relative to 18S)
WT
Akt1ΔECAkt2KO
**
***
Akt1
Akt2

Supplementary Figure 2. Inducible Akt deletion in endothelial cells *in vitro* and *in vivo*. (A-C) Representative confocal images of heart sections (A-low and B-high magnification) and whole mount retinas (C) from adult mT/mG;Akt1ΔEC;Akt2KO mice treated with 5 days of i.p. tamoxifen and 3 weeks of tamoxifen diet (4 weeks tamoxifen treatment) which express mT (red) in all tissues, and mG (green) in cells where tamoxifen-induced Cre recombinase production results in mG expression and Akt1 deletion. Tissues were also stained with CD31 (blue) to visualize endothelial cells and GFP (green) to amplify the mG signal. Quantification of mG labeled CD31+ cells represented as mean±SEM (*n*=4-5). (D) Gene expression of *Akt1, Akt2*, or *CD31* normalized to *18S* in isolated endothelial cells from hearts of tamoxifen treated WT and Akt1ΔEC;Akt2KO mice represented as mean fold change from WT±SEM (*n*=3). (E) Representative immunoblotting of WT, Akt1ΔEC, Akt2KO, and Akt1ΔEC;Akt2KO endothelial cell lysates after 3 days of 60ng/mL VEGF treatment followed by 3 days of 1μM tamoxifen treatment and quantitative results showing fold change in pan-Akt (mean±SEM (*n*=3)) with actin as a loading control. (F) Representative images of matrigel sections from adult WT (*i, ii*) and Akt1ΔEC;Akt2KO (*iii, iiii*) mice treated for 4 weeks with tamoxifen. Sections were stained with Akt1 (green, cytoplasmic or nuclear localization) and CD31 (red, cell junctions, indicated with red arrows) abs. Pictures were taken at the middle of the plugs, where perivascular cells are sparse, therefore, the vast majority of cells are either endothelial or blood cells. Note the presence of Akt1 in blood cells (white arrows) but not in endothelial cells (red arrows show CD31 positive staining at the cell junctions) in Akt1ΔEC;Akt2KO *(iii, iiii*). Bar graph shows quantification of $Akt1^{+}$ /$CD31^{+}$ staining ratio in matrigel sections from WT and Akt1ΔEC;Akt2KO mice (mean±SEM). (G) Percentage of $CD31^{+}$ endothelial cell or non-endothelial total cell content in matrigel plugs containing 250ng VEGF after 7 days of growth isolated from WT or Akt1ΔEC;Akt2KO mice treated with 4 weeks tamoxifen treatment represented as mean±SEM (*n*=4). This demonstrates that only 50% of matrigel plugs are endothelial cells. (H) Gene expression of *Akt1* or *Akt2* normalized to *18S* in matrigel plugs (after 7 days growth with 250ng VEGF) isolated from WT or Akt1ΔEC;Akt2KO mice after 4 weeks of tamoxifen treatment represented as mean fold change from WT±SEM (*n*=3). * represents $p<0.05$, ** represents $p<0.01$, and *** represents $p<0.005$ by one-way ANOVA (E) or Student's *t* test (C,D,G,H).

Supplementary Figure 3

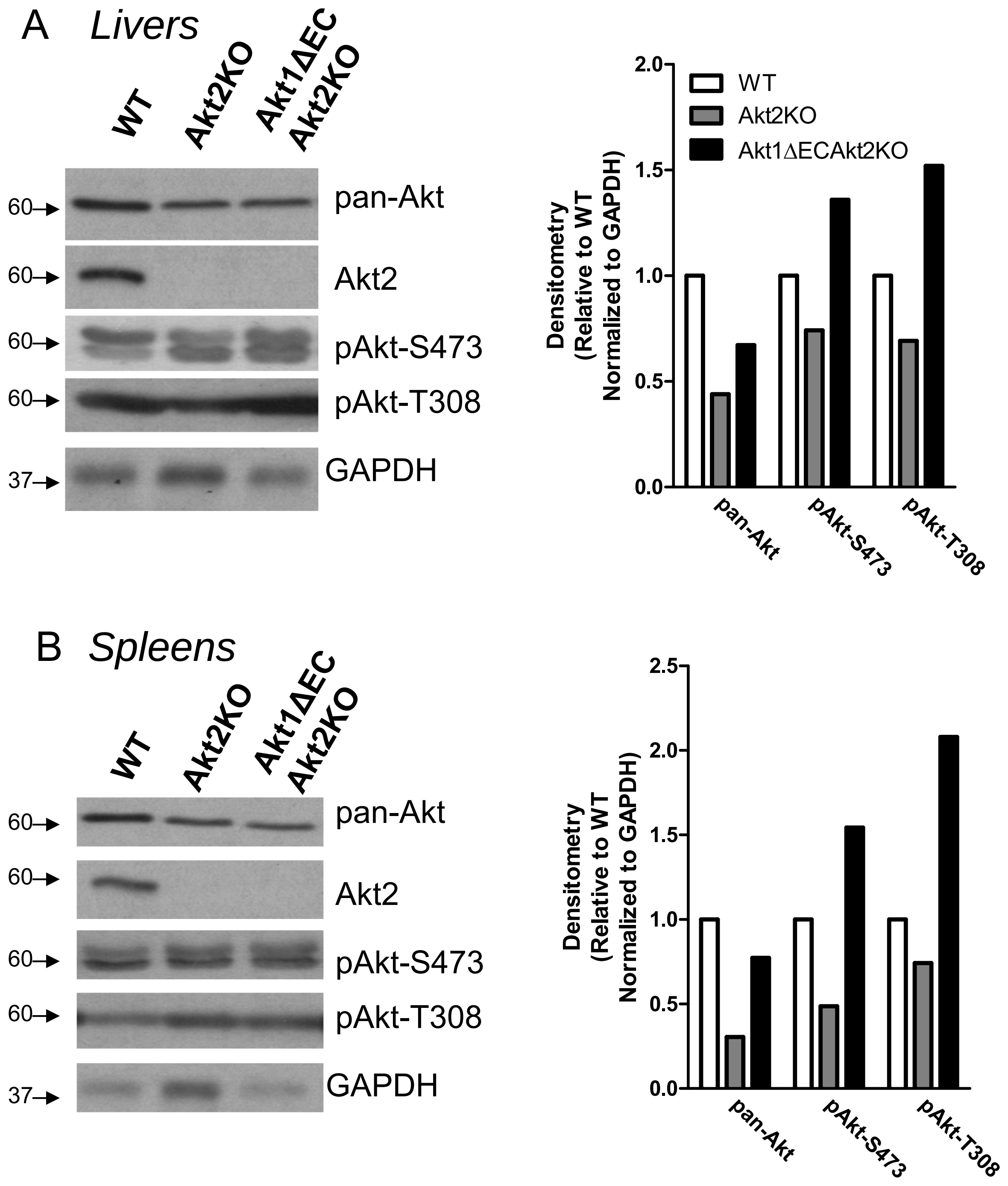

Figure S3

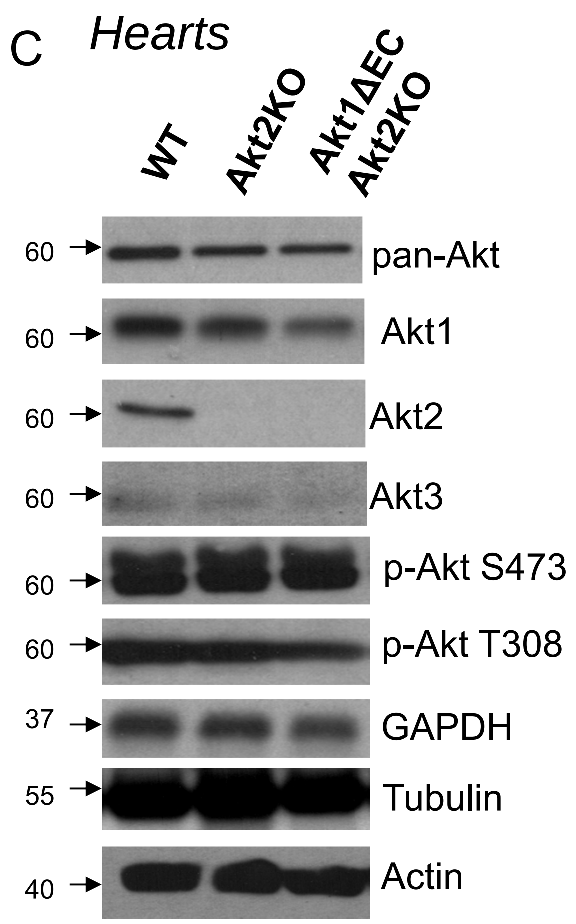
C
Hearts
WT
Akt2KO
Akt1ΔEC Akt2KO
60
pan-Akt
60
Akt1
60
Akt2
60
Akt3
60
p-Akt S473
60
p-Akt T308
37
GAPDH
55
Tubulin
40
Actin

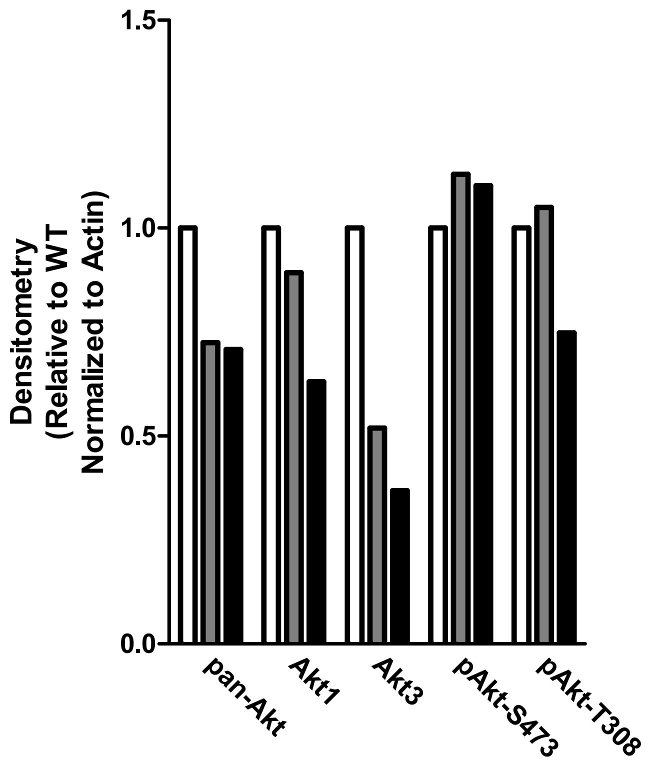
1.5
1.0
0.5
0.0
Densitometry (Relative to WT Normalized to Actin)
pan-Akt
Akt1
Akt3
pAkt-S473
pAkt-T308

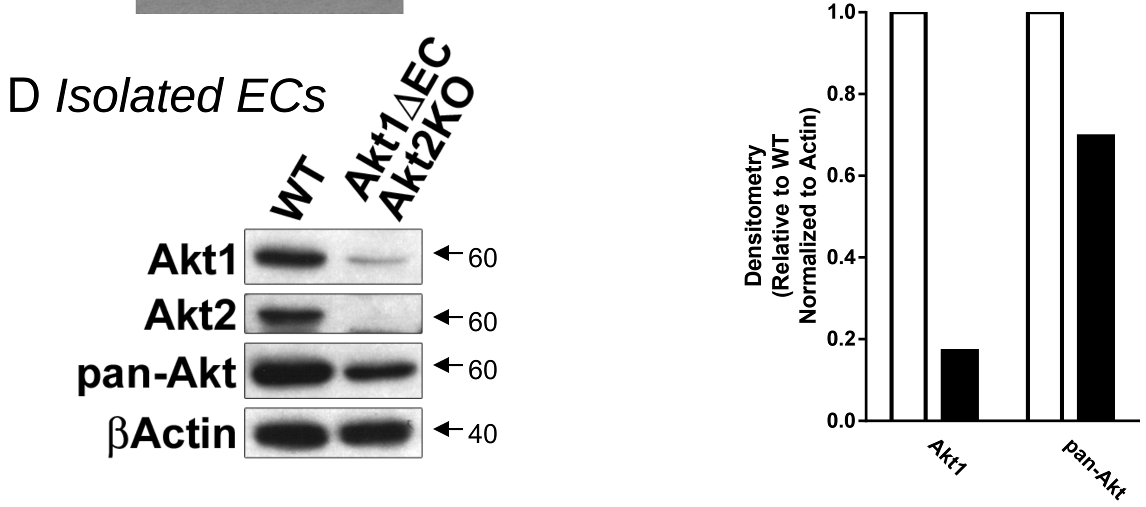
D Isolated ECs
WT
Akt1ΔEC Akt2KO
Akt1
60
Akt2
60
pan-Akt
60
βActin
40
1.0
0.8
0.6
0.4
0.2
0.0
Densitometry (Relative to WT Normalized to Actin)
Akt1
pan-Akt

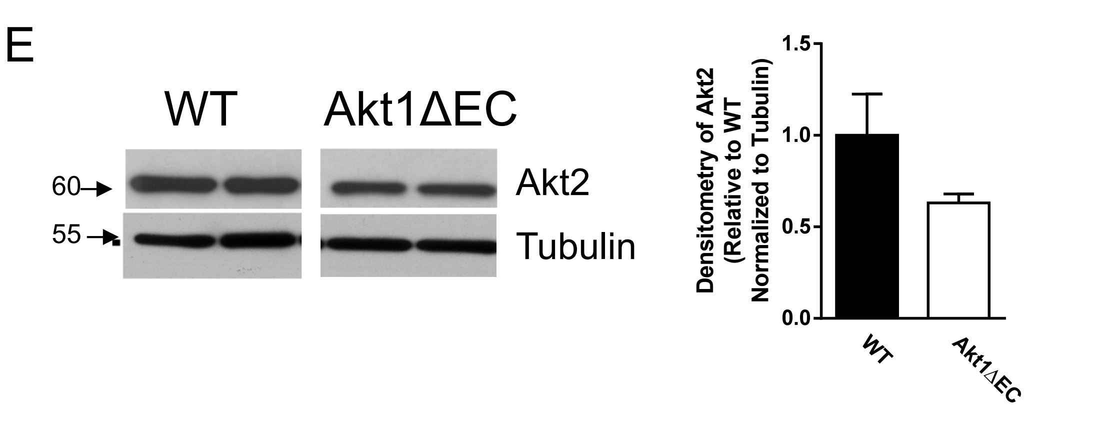
E
WT
Akt1ΔEC
60
Akt2
55
Tubulin
1.5
1.0
0.5
0.0
Densitometry of Akt2 (Relative to WT Normalized to Tubulin)
WT
Akt1ΔEC

Supplementary Figure 3. Deletion of Akt1 in endothelial cells diminishes total Akt levels in hearts, but not liver or spleen. Whole tissue lysates of livers (A), spleens (B), hearts (C) and isolated endothelial cells (D,E) from WT, Akt1ΔEC, Akt2KO, or Akt1ΔEC;Akt2KO mice after 5 days of tamoxifen injection and 3 weeks of tamoxifen diet (4 weeks tamoxifen treatment) were blotted and probed for Akt2, pan-Akt, pAkt-S473, p-Akt-T308, Akt1, Akt2, Akt3, tubulin, actin, and GAPDH. As expected, deletion of Akt1 in endothelial cells affects only small proportion of cells in a given organ and, therefore, does not substantially change total Akt amounts in whole tissue lysates. Indeed, total Akt levels did not change in livers (A), spleens (B), or lungs (not shown). Out of several organs tested, changes in heart lysates were most profound (C). This result likely reflects the relatively higher proportion of endothelial cells in hearts as compared to livers and spleens, as well as the activity of the VE-cadherin (*Cdh5*) promoter. Maximal and consistent decrease in total Akt levels in whole tissue lysates was observed after 5 days of tamoxifen injections followed by tamoxifen diet until completion of experiments.

Supplementary Figure 4

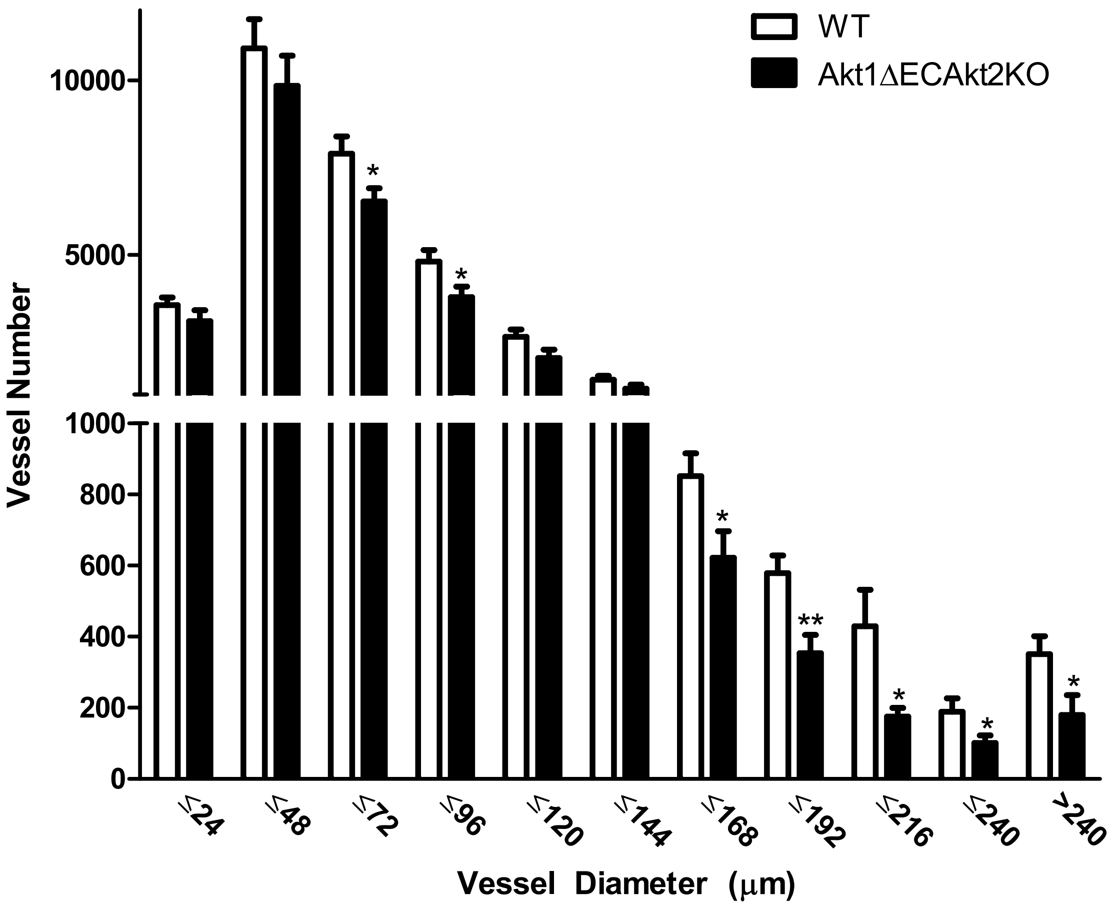

Supplementary Figure 4. Deletion of Akts in hearts diminishes vascularization. MicroCT imaging was performed on WT and Akt1ΔEC;Akt2KO hearts after 10 weeks of tamoxifen treatment (5 days of i.p. tamoxifen injection followed by 9 weeks of tamoxifen diet). Quantification of the numbers of vessels within each diameter bin represented as mean±SEM (*n*=5-7). * represents $p<0.05$ and ** represents $p<0.01$ by Student's *t* test.

Supplementary Figure 5

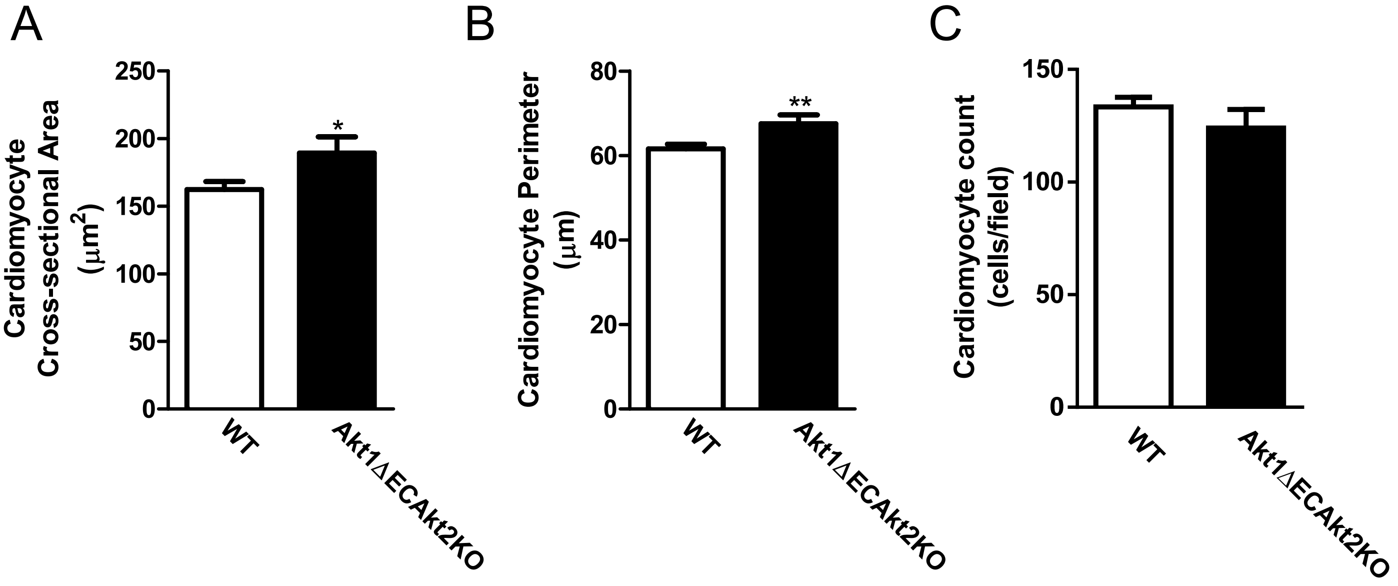

Supplementary Figure 5. Deletion of Akts in hearts has no significant effect on cardiomyocyte numbers. Heart sections from WT or Akt1ΔEC;Akt2KO mice after 5 days of tamoxifen injection and 3 weeks of tamoxifen diet (4 weeks tamoxifen treatment) were stained with wheat germ agglutinin-488. The area, perimeter and numbers of cardiomyocytes in each field were calculated and represented as mean ± SEM (n=9-20). * represents $p<0.05$ and ** represents $p<0.01$ by Student's *t* test.

Supplementary Figure 6

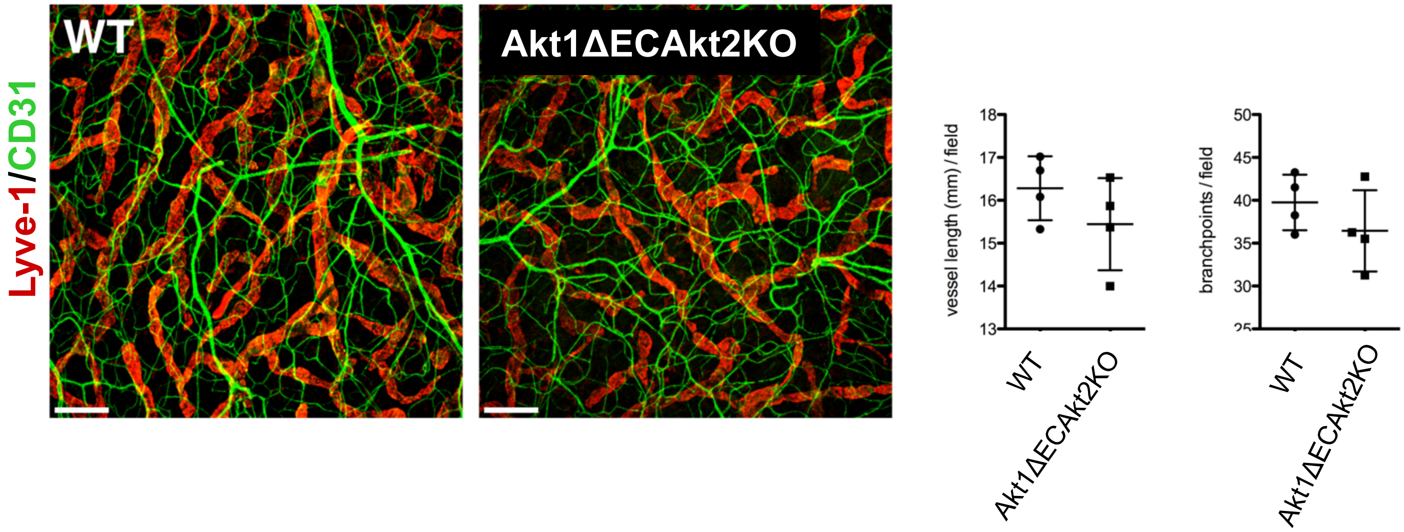

Supplementary Figure 6. Lymphatic vasculature is not significantly decreased after Akt deletion. Ears were isolated from WT and Akt1ΔEC;Akt2KO after 4 weeks of tamoxifen treatment and stained with CD31 (Green) and Lyve-1 (Red). Lymphatic vessel length and branch points per field were quantified and represented as mean±SEM (*n*=4). $p>0.05$

Supplementary Figure 7

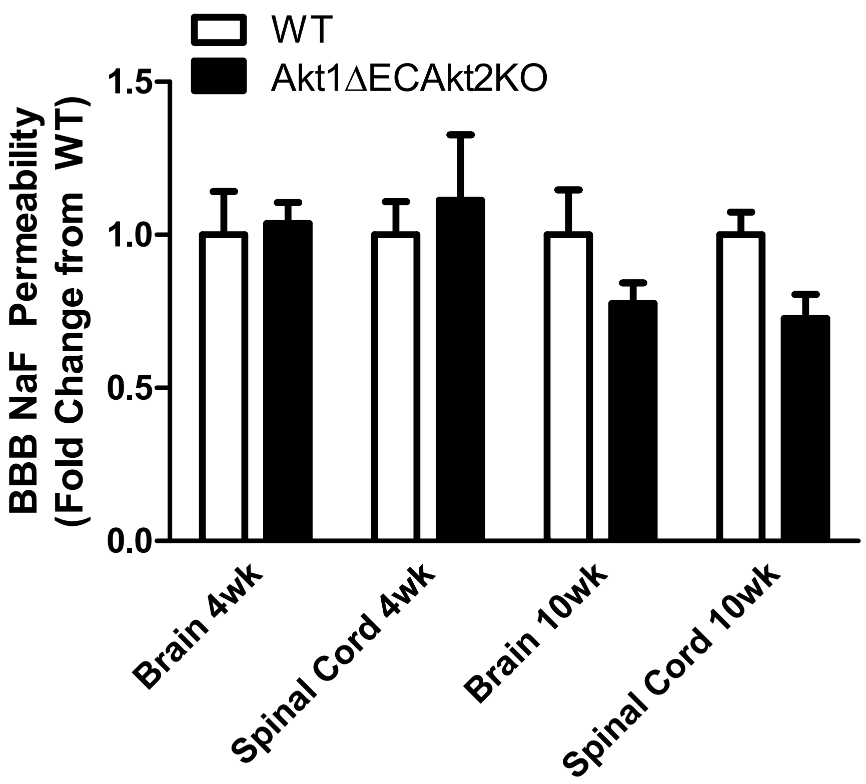

Supplementary Figure 7. Blood-Brain barrier permeability is not altered after Akt deletion. WT and Akt1ΔEC;Akt2KO mice after 4 weeks or 10 weeks of tamoxifen treatment were injected with NaF prior to sacrifice. Brains and spinal cords were isolated and levels of NaF in tissues were quantified and represented as mean±SEM (*n*=4).

Supplementary Figure 8

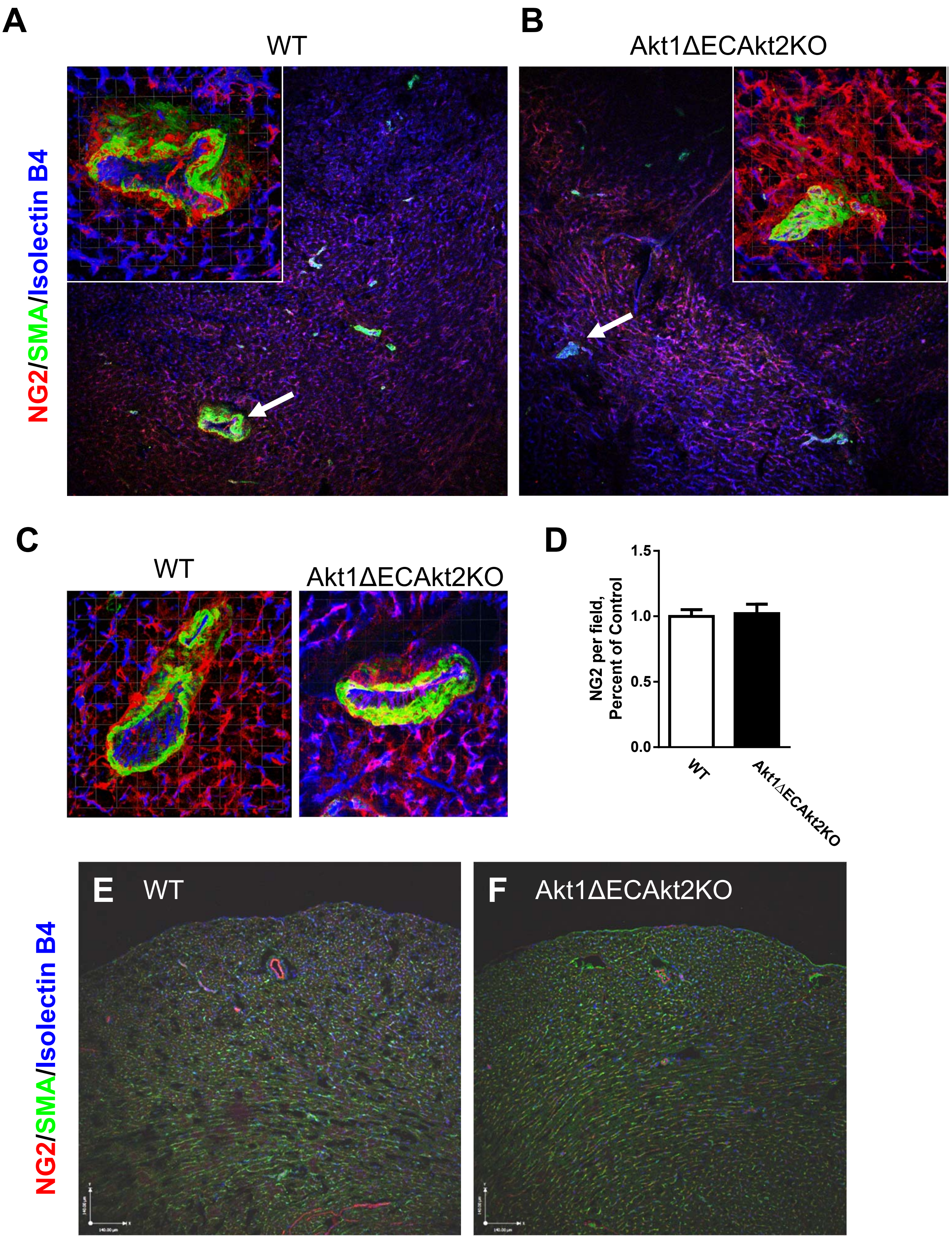

Supplementary Figure 8. Deletion of Akts does not diminish pericyte numbers or pericyte association with endothelial cells. (A-C) Representative images of WT or Akt1ΔEC;Akt2KO heart sections of various thickness, 100 μM (A-B) and 30 μM (C) 10 weeks after tamoxifen treatment stained for isolectin B4 (blue), SMA (green) and NG2 (red). 3D reconstruction was performed using Volocity to observe associations between pericytes and endothelium and the snapshots of 3D images are shown in inserts. No reduction in pericyte numbers was observed in Akt1ΔEC;Akt2KO mice as compared to WT mice. Likewise, no substantial changes in pericyte-endothelial associations were found (Average Pearson coefficients- WT: 0.171, Akt1ΔEC;Akt2KO: 0.204, p=0.13). (D) The area of NG2 positive staining was quantified at 5X (entire cross section of heart) and represented as mean ± SEM (*n*=14). (E,F) Low magnification images showing NG2 staining (green), isolectin (blue) and SMA (red) in cross sections of WT (E) and Akt1ΔEC;Akt2KO (F) hearts, all 10 weeks on tamoxifen.

Supplementary Figure 9

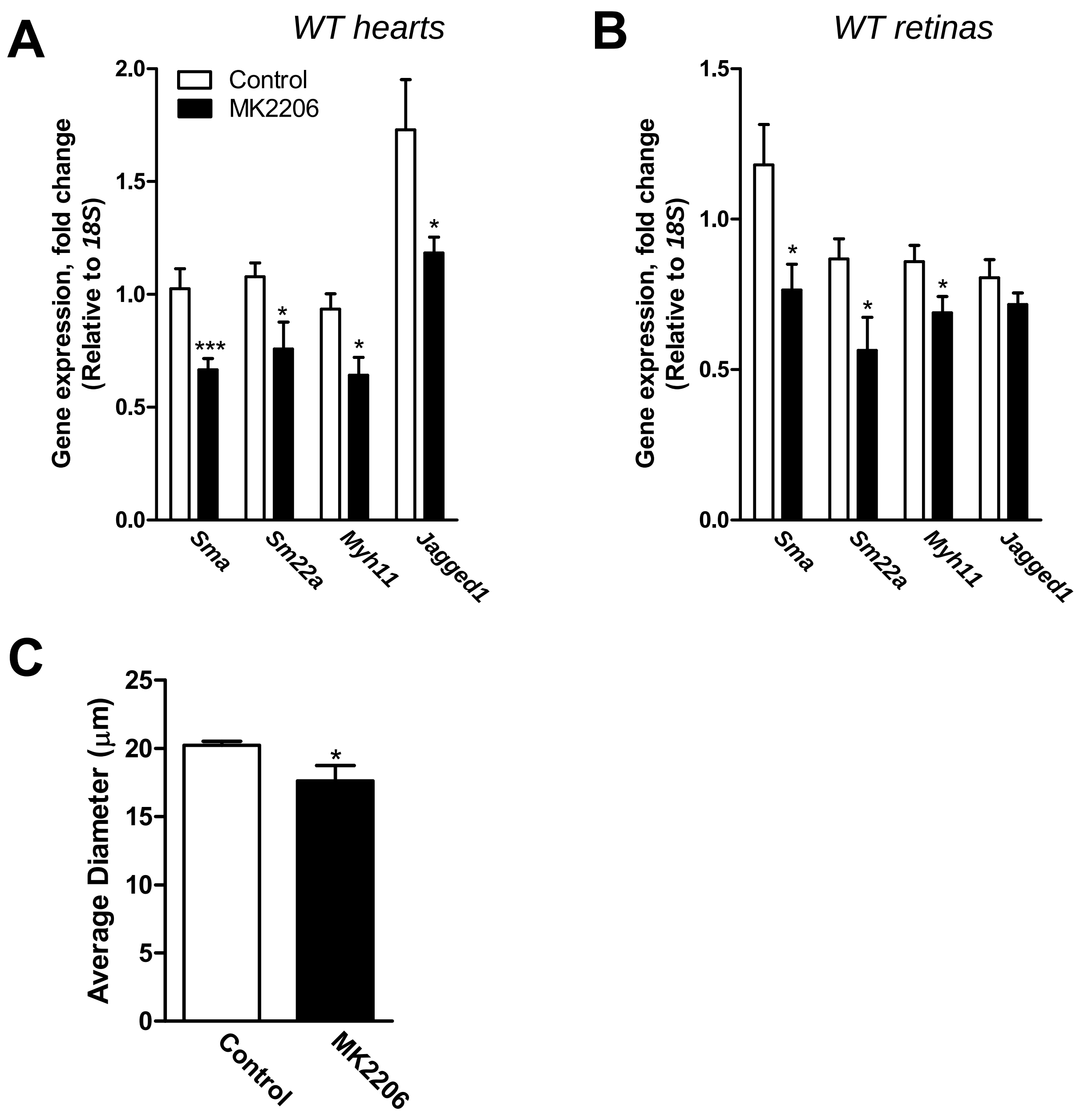

Supplementary Figure 9. Inhibition of Akt by MK2206 in mice resembles the phenotype seen in Akt1ΔEC;Akt2KO. Gene expression normalized to *18S* in WT hearts (A) and retinas (B) from mice after 2 weeks of 120 mg/kg MK2206 or vehicle control (30% captisol) treatment represented as mean fold change from control±SEM (*n*=4). (C) The average diameter of vessels within retinas was calculated and represented as mean±SEM (*n*=3). * represents $p<0.05$ by Student's *t* test.

Supplementary Figure 10

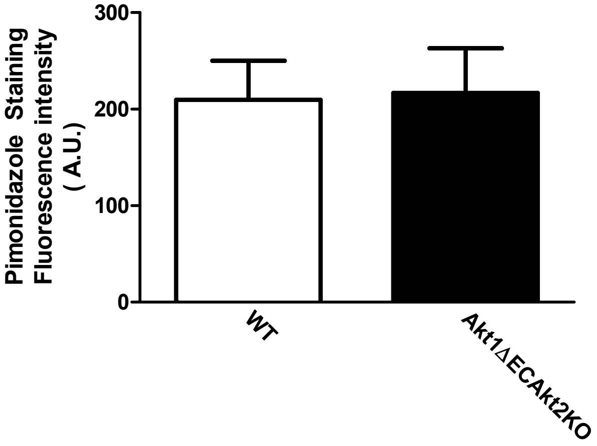

Supplementary Figure 10. Akt deletion has no effect on heart hypoxia. WT or Akt1ΔEC;Akt2KO mice treated with tamoxifen for 10 weeks (5 days of i.p. tamoxifen injection followed by 9 weeks of tamoxifen diet) were injected with 60mg/kg pimonidazole an hour prior to sacrifice. Heart sections were examined by immunofluoresence for pimonidazole and the fluorescence intensity was calculated and mean ± SEM (*n*=4-5).

Supplementary Figure 11

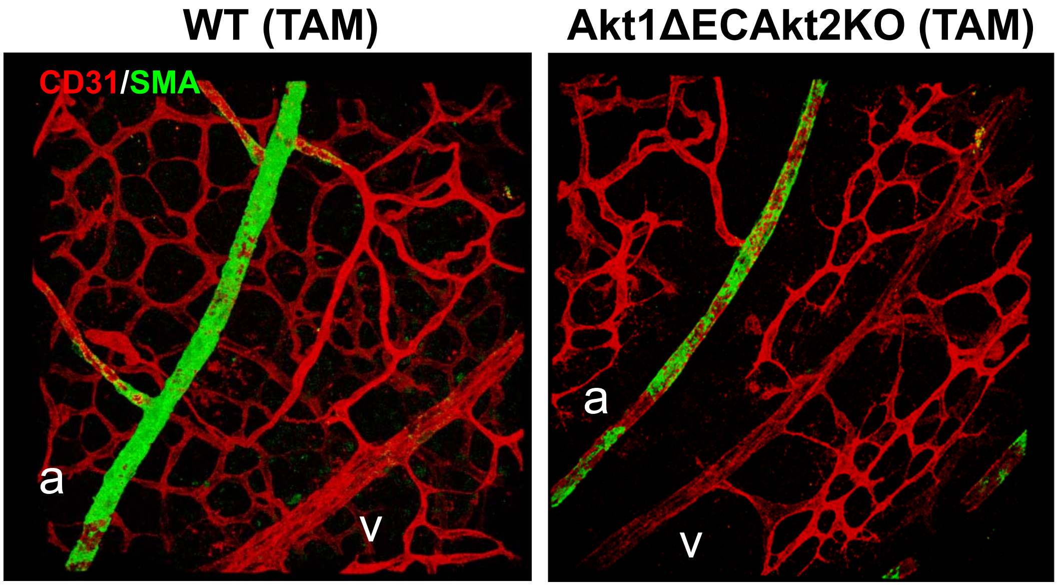

Supplementary Figure 11. Early deletion of Akts results in dramatically reduced VSMC coverage and diminished branching of retinal vasculature. Representative images of whole mount retinas collected at P10 from WT and Akt1ΔEC;Akt2KO mice (*n*=6). Tamoxifen was orally administered starting at P2 for 4 days. Retinas were stained for CD31 (red) to visualize endothelial cells and SMA to visualize VSMC (green).

Supplementary Figure 12

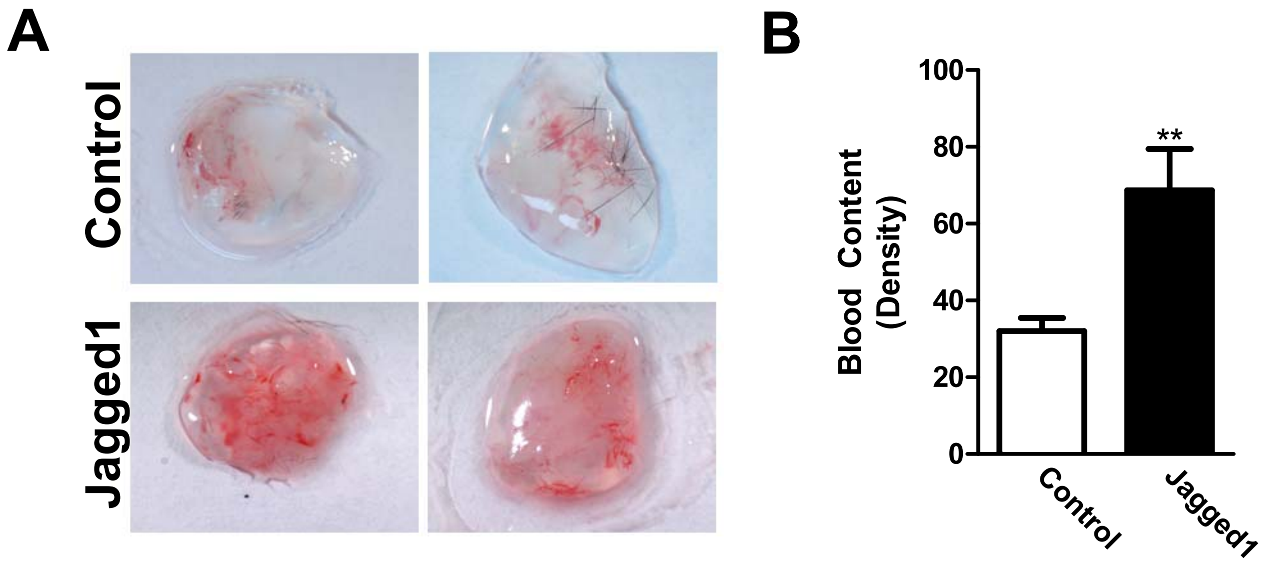

Supplementary Figure 12. Re-expression of Jagged-1 in Akt1ΔEC;Akt2KO endothelial cells improves vascularization and perfusion. (A) Representative images of matrigels containing control or Jagged1 retrovirus infected Akt1ΔEC;Akt2KO ECs after injection into WT mice after 4 weeks tamoxifen treatment. (B) Quantification of perfused vasculature based on blood content is presented as mean density±SEM (*n*=9-10). ** represents $p<0.01$ by Student's *t* test.

Supplementary Figure 13

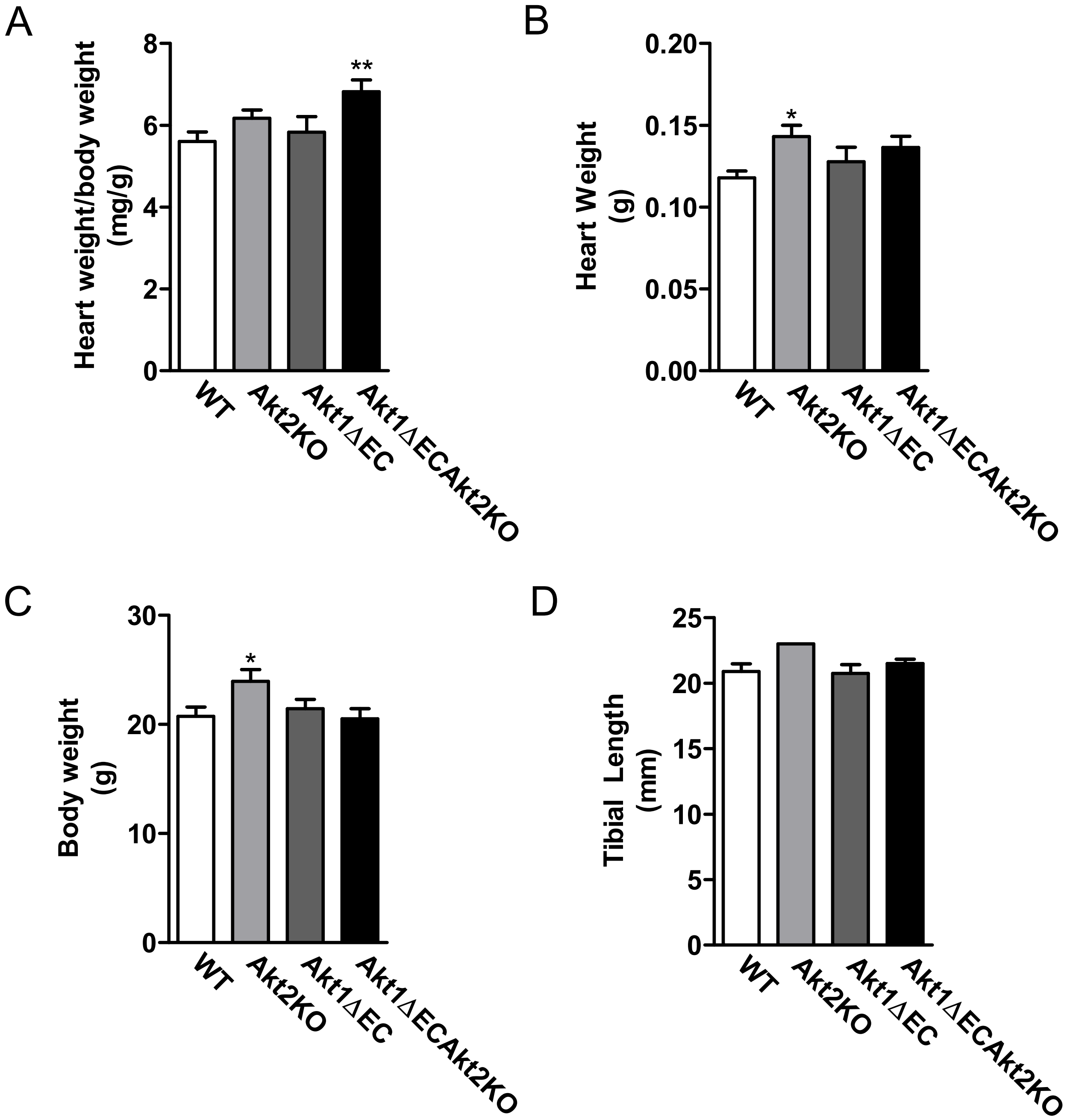

Supplementary Figure 13. Akt deletion in Akt1ΔEC;Akt2KO results in increased heart weight. Heart weight-to-body weight ratios (A), heart weights (B) body weights (C), and tibial lengths (D) of WT, Akt2KO, Akt1ΔEC or Akt1ΔEC;Akt2KO mice treated with tamoxifen for 4 weeks (5 days of i.p. tamoxifen injection followed by 3 weeks of tamoxifen diet). The data are represented as mean ± SEM (*n*=9-20). ** represents $p<0.01$ by one-way ANOVA.

Supplementary Figure 14

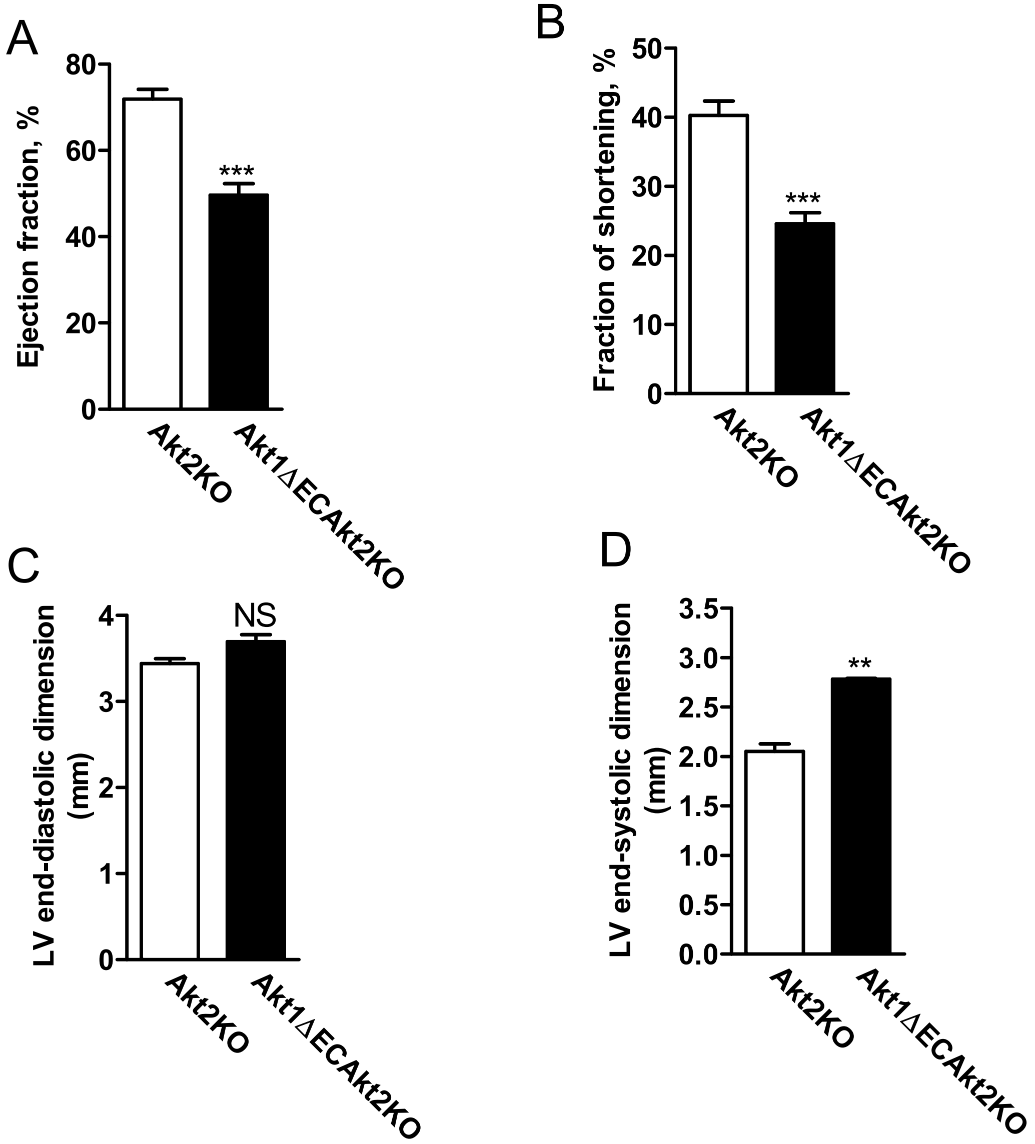

Supplementary Figure 14. Akt1 excision in endothelial cells causes dramatic changes in cardiac function. LVEDD, LVESD, percentage of fractional shortening, and percentage of ejection fraction calculated from echocardiography of Akt2KO and Akt1ΔEC;Akt2KO mice after 10 weeks of tamoxifen treatment (5 days of i.p. tamoxifen injection followed by 9 weeks of tamoxifen diet) representing mean±SEM (*n*=4-6). ** represents $p<0.01$, *** represents $p<0.005$ and NS represents not significant ($p>0.05$) by Student's *t* test.

Supplementary Figure 15

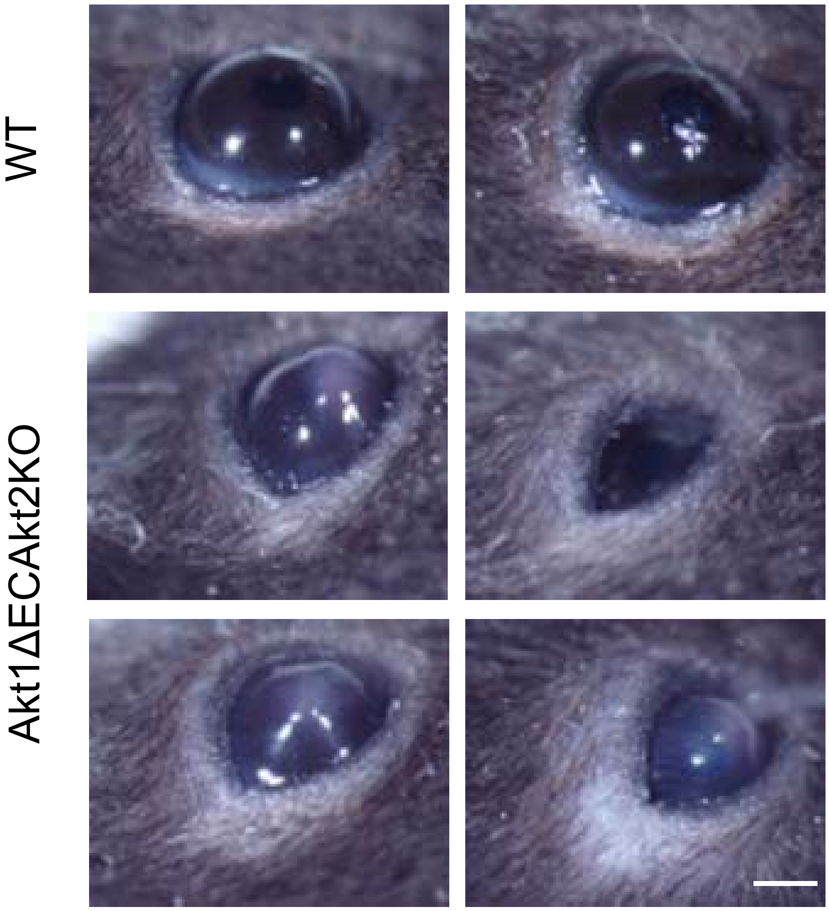

Supplementary Figure 15. Akt deletion in Akt1ΔEC;Akt2KO induces corneal ulcers. Representative images of eyes from WT or Akt1ΔEC;Akt2KO mice treated with tamoxifen for 4 weeks (5 days of i.p. tamoxifen injection followed by 3 weeks of tamoxifen diet). n>50 mice per group. Scale bar represents 1 mm.

Supplementary Figure 16

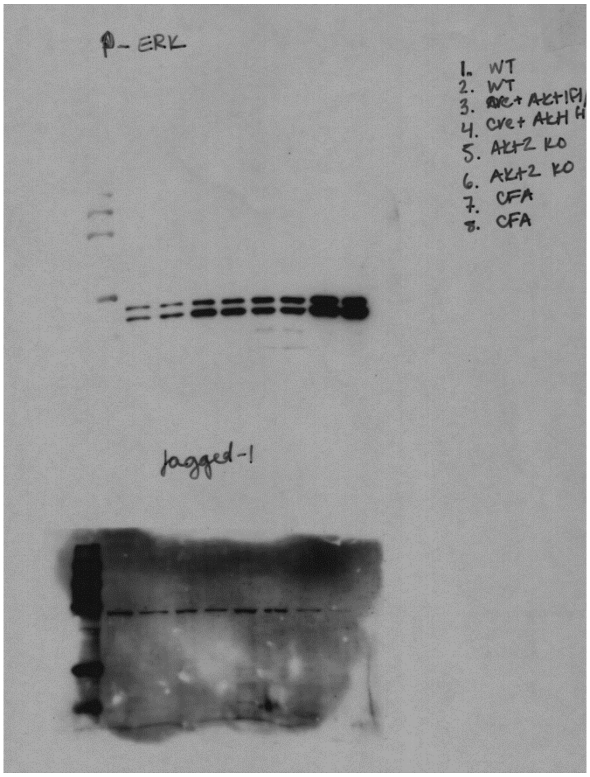

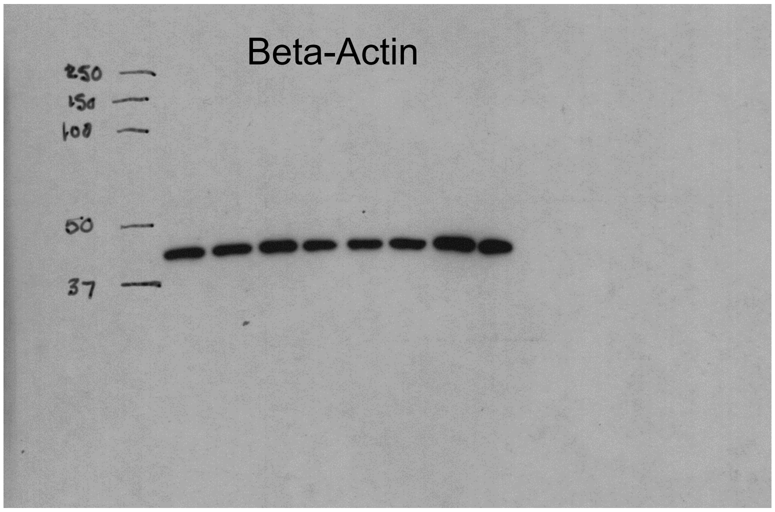

Films of Immunoblots from Figure 5D.

Supplementary Figure 16

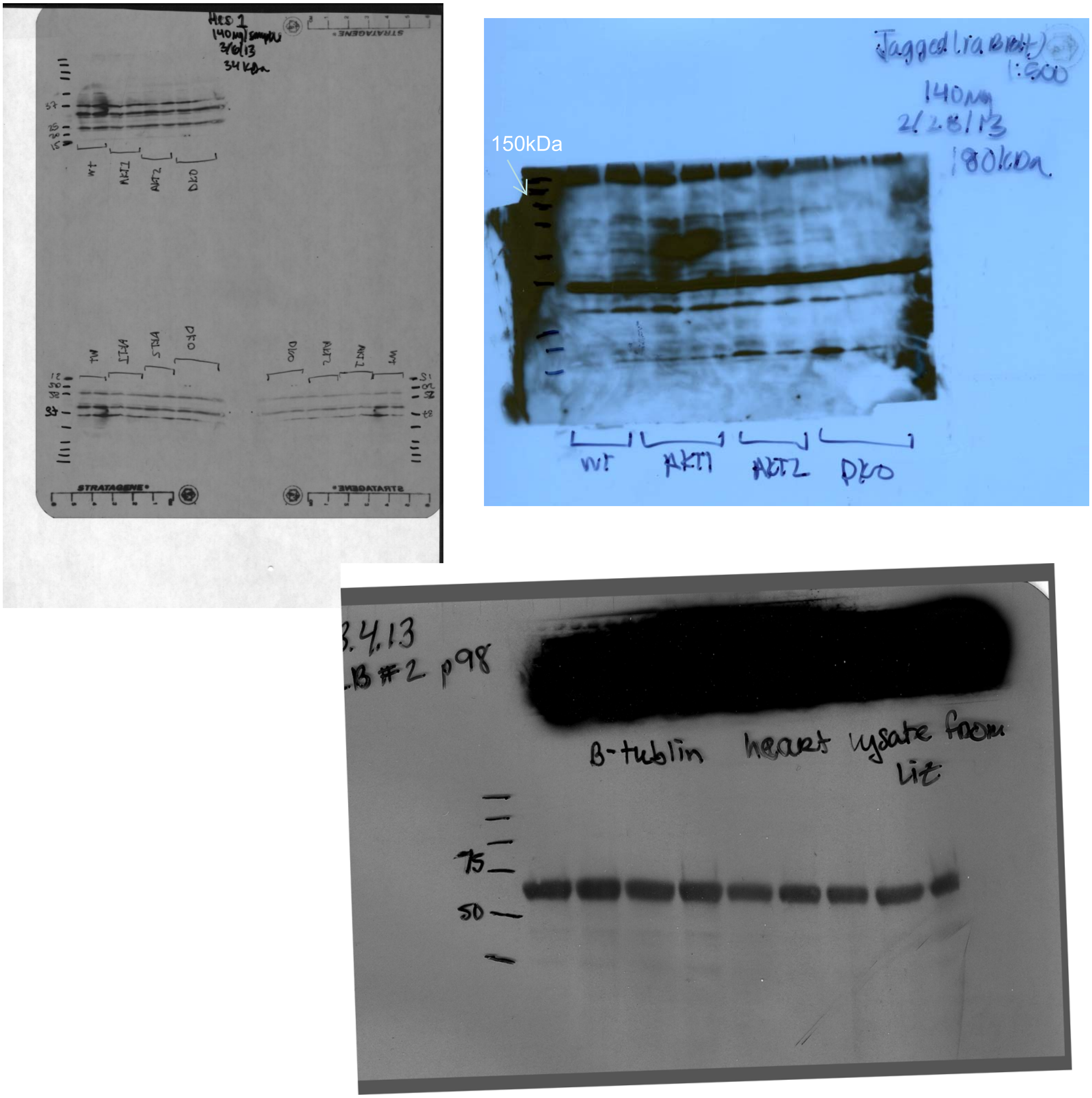

Films of Immunoblots from Figure 5E.

Supplementary Figure 16

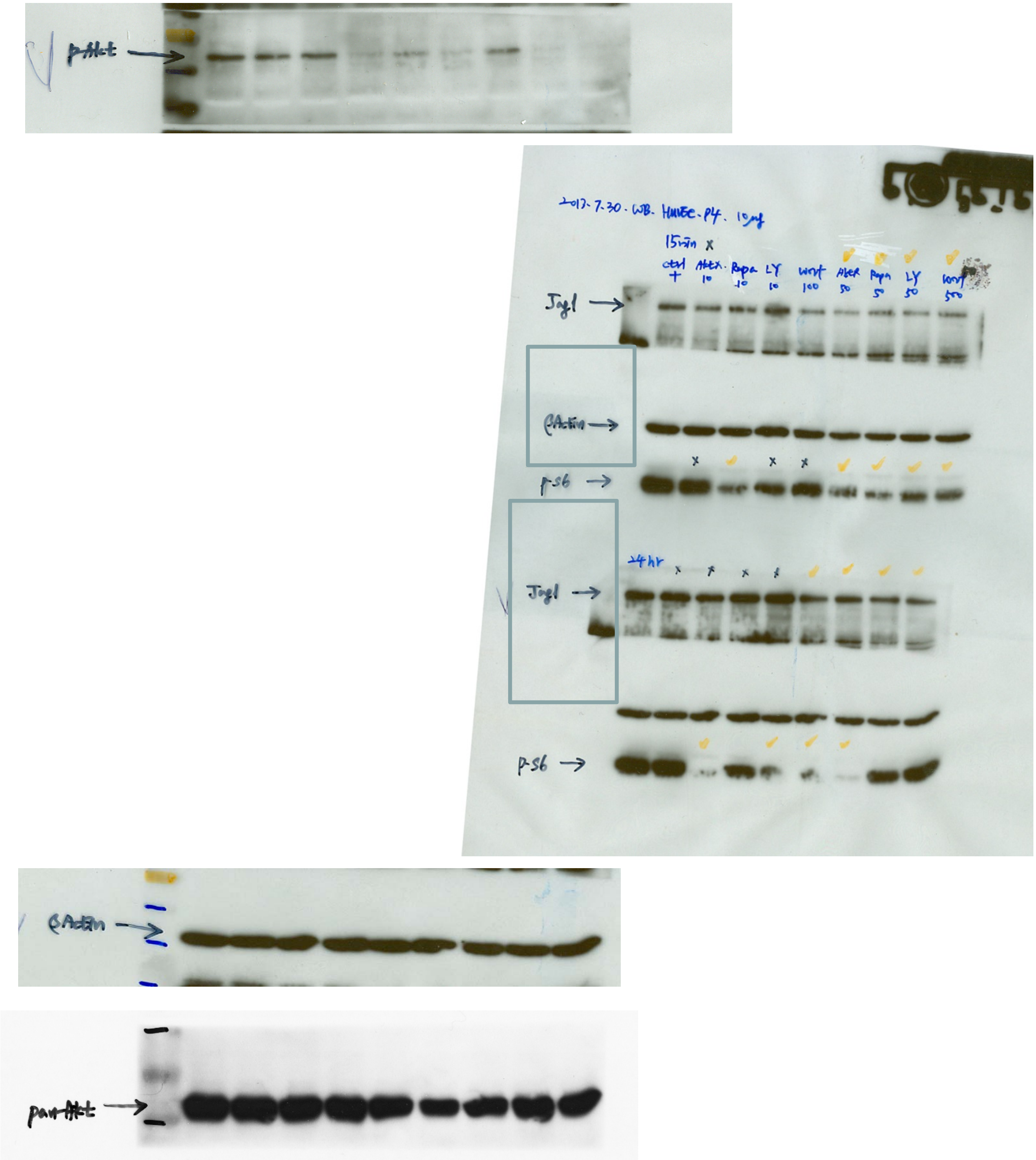

Films of Immunoblots from Figure 5F.

SUPPLEMENTARY TABLE 1

**Increased expression >1.5 fold**	**Decreased expression <-1.5 fold**	**Not Significantly Altered**
Fatty acid binding protein 7 (*Fabp7*)	Brain derived neurotrophic factor (*Bdnf*)	c-Abl (*Abl1*)
FMS-like tyrosine kinase 1; VEGFR1 (*Flt1*)	Core-binding factor, runt domain, alpha subunit 2, translocated to 3 (*Cbfa2t3*)	A disintegrin-like and metallopeptidase with thrombospondin type 1 motif; ADAMTS-1 (*Adamts1*)
Inhibitor of DNA binding 4 (*Id4*)	Cyclin D1 (*Ccnd1*)	Cyclin D2 (*Ccnd2*)
LFNG O-fucosylpeptide 3-beta-N-acetylglucosaminyltransferase; lunatic fringe (*Lfng*)	CREB binding protein; CBP (*Crebbp*)	Circadian locomotor output cycles kaput (*Clock*)
Prostaglandin-endoperoxide synthase 2; Cox-2 (*Ptgs2*)	Chemokine (C-X3-C motif) ligand 1 (*Cx3cl1*)	Chemokine (C-X-C motif) ligand 1 (*Cxcl1*)
Runt related transcription factor 2 (*Runx2*)	Chemokine (C-X-C motif) ligand 12 (*Cxcl12*)	Ephrin A1 (*Efna1*)
Sphingosine phosphate lysase 1 (*Sgpl1*)	Dickkopf homolog 1 (*Dkk1*)	Ephrin B1 (*Efnb1*)
Tenascin C (*Tnc*)	Deltx 1 homolog (*Dtx1*)	Frizzled-related protein (*Frzb*)
Wingless-related MMTV integration site 5A (*Wnt5a*)	Endothelin 1 (*Edn1*)	Frizzled homolog 5 (*Fzd5*)
	Early growth response 3 (*Egr3*)	Heparin-binding EGF-like growth factor (*Hbegf*)
	Four jointed box 1 (*Fjx1*)	Insulin-like growth factor binding protein 3 (*Igfbp3*)
	Forkhead box C1 (*Foxc1*)	Kalirin; Rho GEF kinase (*Kalrn*)
	Forkhead box D3 (*Foxd3*)	MAP/microtubule affinity-regulating kinase 1 (*Mark1*)
	Forkhead box F1a (*Foxf1*)	Nicotinamide phosphoribosyltransferase (*Nampt*)
	Growth arrest and DNA-damage inducible 45 beta; MyD118 (*Gadd45b*)	Pre B-cell leukemia transcription factor 1 (*Pbx1*)
	G-protein signaling modulator 2 (*Gpsm2*)	Platelet derived growth factor receptor, beta polypeptide (*Pdgfrb*)
	Hairy and enhancer of split 1 (*Hes1*)	Recombination signal binding protein for immunoglobulin kappa J region (*Rbpj*)
	Hairy and enhancer of split 5 (*Hes5*)	Rho family GTPase 1 (*Rnd1*)
	Hairy and enhancer of split 7 (*Hes7*)	SNW domain containing (*Snw1*)
	Hairy/enhancer-of-split related with YRPW motif 1 (*Hey1*)	Vascular endothelial growth factor A (*Vegfa*)
	Hairy/enhancer-of-split related with YRPW motif 2 (*Hey2*)	
	Hairy/enhancer-of-split related	

	with YRPW motif-like (*Heyl*)<br>Heat shock protein 8 (*Hspb8*)<br>Inhibitor of DNA binding 1 (*Id1*)<br>Inhibitor of DNA binding 2 (*Id2*)<br>Inhibitor of DNA binding 3 (*Id3*)<br>Interleukin 33 (*Il33*)<br>Jagged1 (*Jag1*)<br>Jun oncogene; c-Jun (*Jun*)<br>Kit ligand; SCF (*Kitl*)<br>Keratin 14 (*Krt14*)<br>Musculin (*Msc*)<br>Myogenic factor 5 (*Myf5*)<br>Nestin (*Nes*)<br>Nodal (*Nodal*)<br>Notch gene homolog 1 (*Notch1*)<br>Notch gene homolog 3 (*Notch3*)<br>Notch-regulated ankyrin repeat protein (*Nrarp*)<br>Paired box gene 6 (*Pax6*)<br>Protocadherin 8 (*Pcdh8*)<br>Platelet derived growth factor, B polypeptide (*Pdgfb*)<br>Platelet derived growth factor receptor, alpha polypeptide (*Pdgfra*)<br>Pre T-cell antigen receptor alpha (*Ptcra*)<br>Recombination signal binding protein for immunoglobulin kappa J region-like (*Rbpjl*)<br>Ras homolog gene family, member V (*Rhov*)<br>Runt related transcription factor 1 (*Runx1*)<br>Sphingosine-1-phosphate receptor 3 (*S1pr3*)<br>Snail homolog 1 (*Snai1*)<br>Suppressor of cytokine signaling	

	3 (*Socs3*)<br>SRY-box containing gene 9 (*Sox9*)<br>Transcription factor 15 (*Tcf15*)<br>Tec protein tyrosine kinase (*Tec*)<br>WNT1 inducible signaling pathway protein 1 (*Wisp1*)<br>Wingless-related MMTV integration site 4 (*Wnt4*)<br>Wingless-related MMTV integration site 6 (*Wnt6*)	

Supplementary Table 1. Genes encoding Notch signaling targets showing altered expression in isolated smooth muscle cells from Akt1ΔEC;Akt2KO mice. Total RNA was isolated from smooth muscle cells using the Qiagen RNeasy kit and cDNA was synthesized using the Qiagen RT^2 First Strand kit. Real-time PCR was performed using Qiagen RT^2 Profiler PCR Array kit for Mouse Notch Signaling Targets and measured on a BioRad myIQ2 iCycler. Data was analyzed by the $\Delta\Delta C_T$ method using the SABiosciences PCR Array Data analysis tools to calculate fold change increased expression or decreased expression in Akt1ΔEC samples compared to WT.

SUPPLEMENTARY TABLE 2

Gene	Forward primer sequence	Reverse primer sequence
mAkt1	gcccaacacctttatcatcc	gtccatcgtctcttcttcctg
mAkt2	ccttccttacagccctcaagt	tgacacaatctctgctccataa
mSma	atcagcaaacaggaatacgac	aaaccacgagtaacaaatcaaa
mCd31	cacacacacacaaagtcaacc	tagccagccatttctcttctt
mCdh5	aaggggcatacagacacaaa	gcttatcacacagcaaacct
mHes1	caaagcctatcatggagaagag	ggaggtgcttcacagtcattt
mHey2	ctccacacatcagagtcaacc	gccgaaagagacagaagagag
hHEY2	agcagtgcattggtataagca	tggagagttttcaagttggca
mJag1	ccgccataggtagagtttga	atttcccagccaaccaca
hJAG1	acaataggatgggcttctgtg	atcacctgcctgtctcttttc
mDll4	aatcatcatccaaggctctct	aaagacatataggctggtcaca
mNotch3	aaccagaagttacccccaag	cacactacaagacagagaggtg
mVegf	ggaggcagagaaaagagaaag	acacacacacacacacacaca
mPdgfrβ	gaccagttttctttccccttt	ccccagagtgtgaagtgtatc
mp63	ctgctcaaaggcacacatc	caacaggaggtaacagaaaagg
mStat3	taaagggcaaaacacactgac	gcatactgggtaaacaaaacc
hSTAT3	tgtctccttgcgtgtctaaag	acaagtgccactggatatcac
18s	gcggttctattttgttggttt	ctccgactttcgttcttgatt
mNotch1	gcaggctccagtgttctgt	agtccaggcagtgttttctct
mNotch4	ggaaggaaggaagcgacac	aggaaaatcacggtctatctca
mMyh11	cgacacagcctacagaagca	tctttcttgcccttgtggga
mSm22a	gctgtgaccaaaaacgatg	gccttccctttctaactgatg

Supplementary Table 2. Primer sequences for genes assayed by quantitative PCR.
